# Factors associated with brain ageing - a systematic review

**DOI:** 10.1186/s12883-021-02331-4

**Published:** 2021-08-12

**Authors:** Jo Wrigglesworth, Phillip Ward, Ian H. Harding, Dinuli Nilaweera, Zimu Wu, Robyn L. Woods, Joanne Ryan

**Affiliations:** 1 School of Public Health and Preventive Medicine, Monash University, Melbourne, Victoria 3004 Australia; 2 Monash Biomedical Imaging, Monash University, Clayton, Victoria 3168 Australia; 3 Turner Institute for Brain and Mental Health, Monash University, Clayton, Victoria 3800 Australia; 4 Australian Research Council Centre of Excellence for Integrative Brain Function, Clayton, Victoria 3800, , Australia; 5 Department of Neuroscience, Central Clinical School, Monash University, Melbourne, Victoria 3004 Australia

**Keywords:** Brain ageing, BrainAGE, Predicted age difference, Age prediction, Neuroimaging, Machine learning, Biomarker, Age-related brain changes

## Abstract

**Background:**

Brain age is a biomarker that predicts chronological age using neuroimaging features. Deviations of this predicted age from chronological age is considered a sign of age-related brain changes, or commonly referred to as brain ageing. The aim of this systematic review is to identify and synthesize the evidence for an association between lifestyle, health factors and diseases in adult populations, with brain ageing.

**Methods:**

This systematic review was undertaken in accordance with the PRISMA guidelines. A systematic search of Embase and Medline was conducted to identify relevant articles using search terms relating to the prediction of age from neuroimaging data or brain ageing. The tables of two recent review papers on brain ageing were also examined to identify additional articles. Studies were limited to adult humans (aged 18 years and above), from clinical or general populations. Exposures and study design of all types were also considered eligible.

**Results:**

A systematic search identified 52 studies, which examined brain ageing in clinical and community dwelling adults (mean age between 21 to 78 years, ~ 37% were female). Most research came from studies of individuals diagnosed with schizophrenia or Alzheimer’s disease, or healthy populations that were assessed cognitively. From these studies, psychiatric and neurologic diseases were most commonly associated with accelerated brain ageing, though not all studies drew the same conclusions. Evidence for all other exposures is nascent, and relatively inconsistent. Heterogenous methodologies, or methods of outcome ascertainment, were partly accountable.

**Conclusion:**

This systematic review summarised the current evidence for an association between genetic, lifestyle, health, or diseases and brain ageing. Overall there is good evidence to suggest schizophrenia and Alzheimer’s disease are associated with accelerated brain ageing. Evidence for all other exposures was mixed or limited. This was mostly due to a lack of independent replication, and inconsistency across studies that were primarily cross sectional in nature. Future research efforts should focus on replicating current findings, using prospective datasets.

**Trial registration:**

A copy of the review protocol can be accessed through PROSPERO, registration number CRD42020142817.

**Supplementary Information:**

The online version contains supplementary material available at 10.1186/s12883-021-02331-4.

## Introduction

Ageing is a complex biological process characterised by an accumulation of molecular and cellular damages over the lifespan [1–3]. The body’s inability to repair this damage leads to a subsequent loss of physiological functions [1]. These include sensory, motor, and cognitive functions that, when impaired, impact quality of life [4]. Age is also a major risk factor for many life threatening diseases including cancer, cardiovascular disease, and neurodegenerative disorders [1]. The trajectory of ageing, however, varies within the population, and thus, chronological age is not always a reliable predictor of age-related risk. Genetic and environmental factors are diverse among the population, and have varied effects on ageing processes occurring within individual cells, and tissue types [2].

The brain is particularly sensitive to the effects of ageing, manifesting as changes in structure and cognitive function [5–8]. Neuroimaging technologies, including magnetic resonance imaging (MRI), have made it possible to monitor these changes in vivo. The most common changes associated with ageing are brain atrophy (i.e., loss of grey matter volume and cortical thinning) [9–12], a reduction in white matter integrity and volume, and abnormal functional connectivity [7, 13–16]. When severe, these phenotypes can be considered a sign of accelerated ageing or an underlying disease process [5, 6]. Though neuroimaging research has advanced our understanding of these processes, current group based analyses (i.e., mass univariate modelling that uses chronological age to predict neuroimaging features), cannot account for the diversity of individual ageing trajectories [17].

Among these developments are efforts focused on identifying individual biomarkers of age-related brain changes [18]. So-called 'brain age' algorithms use neuroimaging features to capture the changes in the brain that commonly occur with age [18]. Typically, this requires training a multivariate statistical model to learn normative patterns of brain ageing, before being applied to predict individual brain ages in a group of interest. The difference between predicted biological and actual chronological age signifies a deviation from the normal ageing trajectory, and has the potential to identify individuals with disease, monitor treatment effects, or identify lifestyle factors that are beneficial or detrimental to brain health [18–20].

A recent literature review summarised different methods that use brain volume to define brain age [20]; whilst another provided a more comprehensive overview of all methodologies currently being applied in the field, including developmental and animal studies [21]. However, to date, no systematic review has summarised age-related brain changes (referred to as ‘brain ageing’), defined solely by the deviation of estimated brain age from chronological age, in human adult populations. Thus, the aim of this systematic review is to identify and synthesize the evidence for an association between lifestyle, health factors, and diseases in adult populations, with brain ageing.

## Methods

### Protocol and registration

This systematic review was undertaken in accordance with the PRISMA guidelines (http://www.prisma-statement.org) - the 2009 checklist is provided in Additional File 1 [22]. In compliance with these guidelines, a record of this protocol can be accessed through PROSPERO via the following registration number CRD42020142817.

### Eligibility criteria

This systematic review included studies investigating brain ageing in adult humans (mean age 18 years and above), from community or clinical populations. Studies measured exposures of all types, including genetic, health, and lifestyle factors, and the outcome was brain ageing. All study designs (cohort and case-control) were eligible, with brain ageing measured either at the same time as the exposure (cross sectionally) or a later time-point (longitudinally). Papers limited to evaluating the sensitivity of different methodologies (e.g. sample size) on brain ageing were not included.

### Brain ageing

Estimates of brain age were considered eligible when chronological age was predicted from neuroimaging features, acquired from any imaging modality (e.g., MRI). Eligible studies were those which examined brain ageing as the difference between brain age and chronological age. Studies using alternative methods for calculating brain ageing, including the slope between chronological age and brain age [23]; or the group differences in models of brain features as a function of age [24], were excluded.

### Information sources and search strategy

A systematic search of Embase via Ovid (1974 to present) and Ovid MEDLINE was conducted to identify relevant articles, using search terms relating to the prediction of age from neuroimaging data or brain ageing: (BrainAge.mp. OR Neuroanatomical adj3 age.mp. OR brain age.mp. OR age adj3 estimat*.mp. AND Imaging.mp) OR (BrainAge.mp. OR Neuroanatomical adj3 ag*.mp. OR age adj3 estimat*.mp OR brain ag*.mp. OR BrainAGE adj3 accelerat*.mp OR brain age gap.mp OR BrainPAD.mp OR Brain adj1 predict*.mp AND imaging.mp. AND chronological age.mp. AND accelerat* adj3 ag*.mp). No yearly limit was set, however searches were limited to studies only including human participants, and articles published in English. The tables of two recent review papers on brain ageing [20, 21] were also examined to identify additional articles.

### Study selection

Following the initial search, duplicate articles were removed by one reviewer (JW). Article abstracts and titles were screened independently by three reviewers (JW, DN, ZW), followed by a full text review of the eligible texts. In the case of discordance, a fourth reviewer (JR) was involved to provide a final verdict.

### Data extraction

For each included study, the following information was extracted onto a standardised data extraction form: Study characteristics (i.e., name, country and design); Participant characteristics (i.e., sample size, mean age and/or range, number of female participants); neuroimaging features used for brain age prediction (i.e., modality, protocol, and features) and statistical methodologies (i.e., algorithm, and cross validation, and adjustment for age bias); and exposures (e.g., cognitive function, disease type). Main findings and details of any adjustments for confounders were also extracted.

### Data synthesis/summary measures

A narrative synthesis of the main brain ageing findings is provided, and grouped according to the type of exposure. Findings are summarised quantitatively in tables with effect sizes (when available), regardless of statistical significance. Effect sizes of all types are reported, and include correlations; differences in mean brain ageing (including Cohens D/Eta squared); 95% confidence intervals (when *p*-value was not available), and beta values (both un/standardized) from regression models. Authors considered brain ageing methodologies, and/or participant characteristics too heterogenous to conduct a meta-analysis.

### Risk of bias

Included articles were assessed for risk of bias using a modified version of the Joanna Briggs Institute Critical Appraisal Checklist for Randomized Control Trial, Case-Study or Cohort study, as appropriate [25]. This assessment was merely a tool for determining the quality of information extracted from each article, rather than a means for excluding papers. This was completed by three reviewers (JW, ZW, DN), independently. Any discrepancies were discussed and resolved through consensus.

## Results

### Study selection

**Fig. 1:**
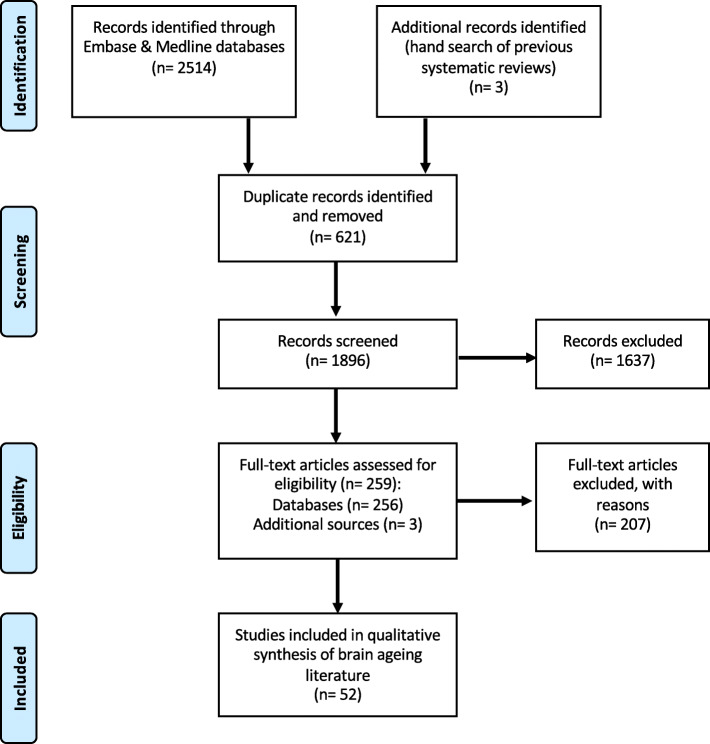
PRISMA flow diagram outlining results from the initial search, and subsequent screening for article eligibility

An initial search of Medline and Embase resulted in 2514 articles, and an additional three papers were identified from prior reviews on brain ageing (Fig. 1) [20, 21]. After removing duplicates, the titles and abstracts of 1896 articles were screened, and 1637 papers excluded. Two hundred and fifty-nine papers underwent a full text review. From these papers, a further 207 articles were removed as they did not meet the eligibility criteria (ineligible article type; sample of children/adolescents only; or ineligible calculation of age prediction). A total of 52 papers were thus included in this systematic review.

### Participant characteristics

Studies investigated brain ageing in samples ranging in size (between 5 to 31,227 participants), and age (mean age between 21 to 78 years). One study compared one male with Prader-Willi syndrome to a small sample of 95 healthy controls (approximately 39% were male) [26]. Four studies included children, and/or adolescents as well as adults, but fit the inclusion criteria given that the mean age of the sample was 18 years or older [27–30]. All but two studies included both men and women, with the percentage of women ranging from 4.4 to 89.1%. Five of these studies, however, did not report the number of men or women [30–34]. Of the two remaining studies, one involved military serving male twins [35], and a second focused on brain ageing in post-menopausal women [36].

Twenty-nine studies sub-sampled participants from a larger cohort study, nine were case-controls [26, 30, 37–43]. Of the remaining 10 case-control studies, eight had sampled participants from registries, hospitals (i.e., both in and outpatient services) or treatment clinics, university research institutes, or the local community [29, 44–50], while two were unclear [51, 52]. The Early Stages of Schizophrenia study [38, 41], the UK Biobank [19, 32, 53, 54] and the Alzheimer’s Disease Neuroimaging Initiative (ADNI) [33, 55–58] were cohorts sampled on more than one occasion. Thirteen studies included prospective data [28, 30, 31, 33, 35, 36, 47, 56, 58–62].

One study estimated brain age for participants who were a part of a randomised control trial [63]. Six studies pooled data from multiple studies [26, 30, 60, 64–66]; while three studies involved more than one type of study design [30, 41, 47].

### Summary of brain ageing findings

Brain ageing was investigated in relation to a number of exposures. These are summarised in the following text and tables, and are grouped according to the type of exposure. ‘Accelerated’ and ‘decelerated’ are terms commonly used to describe the direction of brain ageing (i.e., accelerated defines greater age-related changes to the brain; while decelerated suggests fewer changes) and thus will be used in the subsequent text. Similarly, in longitudinal studies, the ‘rate’ is conventionally used to define a change in brain age, but can be calculated by either regressing time on brain age, or dividing change in brain age by the time interval between the imaging acquisitions. Thus, while rate will be used throughout the following text, methods will be defined in tables accordingly.

In tables, brain ageing (i.e., brain age – chronological age) was abbreviated as the “brain age gap (GAP)”, and used to summarise results. Though conceptually the same, two studies subtracted brain age from chronological age, and thus, “CA-BA” is used to report these results [27, 64]. When studies involve a common brain age framework (i.e., was referenced by more than one study), terms specific to this framework will be used. These include the “Brain age gap estimate (BrainAGE) score” [55], “Predicted age difference (PAD) score” [51], and “Brain ageing (BA) score” [67], and are specific to these referenced authors.

#### Psychiatric disorders

Thirteen studies investigated brain ageing in psychiatric disorders [27, 30, 32, 34, 37, 38, 41, 44, 49, 50, 60, 66], eight focused on schizophrenia (SZ) [27, 30, 32, 34, 38, 41, 49, 66] (Table 1). All studies report accelerated brain ageing in SZ (ranging between 2.3 and 7.8 years), though the majority included samples less than 100 participants. Of these studies, six found accelerated brain ageing to be significantly different to healthy controls [32, 34, 38, 41, 49, 66]; while two made no statistical comparison between groups [27, 30]. Five studies also included patients with bipolar disorder. Four of these found brain ageing to be comparable to healthy controls [32, 34, 41, 49]. The fifth study only reported accelerated brain ageing, and made no statistical comparison to a control group [27].

**Table 1:** Studies investigating the association between mental health and behavioural disorders

Reference	Study (Design, country)	n, Mean age ± SD (Range), Sex, Other information	Modality (Protocol)	Features	Model (Cross-validation)	Exposure	Main findings outcome	Adjustments
[27]	Multisite psychiatric database (Retrospective; US)	SZ: *n* = 657, 30.5 ± 13.7 yrs.; ADHD: *n* = 1462, 21.2 ± 15.5 yrs.; MDD: *n* = 4753, 38.4 ± 17.0 yrs.; BD: *n* = 2524, 31.9 ± 16.1 yrs.; CAD: *n* = 1089, 26.6 ± 10.4 yrs.; AUD: *n* = 1457, 38.4 ± 14.6 yrs.; GAD: *n* = 6457, 32.6 ± 17.3 yrs.; Sex unknown	SPECT (rs-99mTc-HMPAO)	Regional cerebral perfusion	LR	Psychiatric co-morbidities	↑ CA-BA in SZ (−4.0 yrs), CAD (− 2.8 yrs) BD (− 1.7 yrs), ADHD (− 1.4 yrs), AUD (− 0.6 yrs) & GAD (− 0.5 yrs); ↓ in MDs (0.85 yrs)	None
[44]	In- or out-patient services & matched controls (Germany)	MDD: *n* = 38, 45.7 ± 15.7 (19–66) yrs., 21♀; HC: *n* = 40, 42 ± 13.2 (21–73) yrs., 20♀	MRI (T1[3T])	Voxel-wise GM volume	RVR	MDD without axis I/II co-morbidity	NS BrainAGE	^a^Scanner
[43]	Cases and controls from multiple ENIGMA MDD cohorts (Spain, Germany, UK, US, Canada, AUS, Brazil)	MDD: *n* = 2675, 43.08 ± 14.0 yrs., 1689 ♀; HC: *n* = 2126, 40.99 ± 15.82 yrs., 1199 ♀	MRI (T1[1.5/3 T])	CT, SA, subcortical GM volume, lateral ventricles, ICV	RR [Data splitting & 10-fold]	MDD & clinical characteristics	GAP ↑ in MDDs (b = 1.08, *p* < 0.0001). ↑ in first episode (b = 1.22), recurrent depression (b = 0.97), remittance (b = 2.19), current MDD (b = 1.47), AD use (b = 1.36), AD free (b = 0.67), early, mid & late age of onset (~b = 0.91–1.21) respectively (all *p* < 0.05). NS with severity	^b^Age, age^2^, sex, site
[60]	Community dwelling adults from 1 of 6 studies (US)	*n* = 185, 64.9 ± 8.3 yrs., 91♀	MRI (T1[3T])	Voxel-wise WB volume	RVR	Depression	↑ BrainAGE (*r* = 0.23, *p* = 0.01)	^b^Age, gender, diabetes duration
[37]	Cases & matched controls from LeAD study (Germany)	HC: *n* = 97, 43.7 ± 10.8 (21–65) yrs., 16♀; AlcD: *n* = 119, 45.0 ± 10.7 (20–65) yrs., 18♀	MRI (T1[3T])	Cortical & subcortical GM volume	MRR [LOO]	AlcD; lifetime alcohol consumption	60-69 yr AlcD GAP 11.7 yrs. ↑ than HCs (*p <* 0.01). NS in AlcD < 39 yrs. 71 standard drinks correspond to approximately ½ day of GAP in AlcD (β = 0.56, *p* = 0.03)	^a^Gender, site, smoking, LC, general health; ^b^Age
[32]	Icelandic dataset (Iceland)	HC: *n* = 291; SZ: *n* = 68; ID: *n* = 6; ASD: *n* = 10; BD: *n* = 31; Age & sex unknown	MRI (T1[1.5 T])	Voxel-wise MNI, Jacobian map, GM & WM volume	CNN [Data splitting]	SZ, ID, ASD, & BD	SZ GAP 2.2 yrs. ↑ than HC (2.3y vs. 0.1 yrs.; ***p*** **< 0.01**). **NS** for ID, ASD, & BD	^a^Age, sex, TICV
[38]	Cases from the Early Stages of Schizophrenia; community dwelling controls (Czech Republic)	FEP: *n* = 120, 27.0 ± 4.9 (18–35) yrs., 46♀; HC: *n* = 114, 25.7 ± 4.0 (18–35) yrs., 51♀	MRI (T1[3T])	Voxel-wise WB volume	RVR	FEP	FEP directly associated with BrainAGE (B = 1.15 yrs., *p <* 0.01)	^b^Age
[41]	1) Cases from the Early Stages of Schizophrenia & matched controls (Czech Republic); 2) HR offspring from ORBIS, & controls from similar SES (Canada, Prague)	1) FEP: *n* = 43, 27.1 ± 4.9 yrs., 17♀; HC: *n* = 43, 27.1 ± 4.4 yrs., 17♀; 2) HR: *n* = 48, 20.9 ± 4.2 yrs., 29♀; Early BD: *n* = 48, 23.1 ± 4.5 yrs., 33♀; HC: *n* = 60, 23.4 ± 2.9 yrs., 36♀	MRI (T1[1.5/3 T])	Voxel-wise WB volume	RVR	FEP; HR & early BD	1) FEP BrainAGE 2.64 yrs.; HC −0.01 yrs. (Cohens D = 0.64, ***p*** **= 0.008**); 2) HR & early staged BDs comparable to HC (− 0.96, −1.02 yrs. & 0.25 yrs., respectively; **NS**)	^b^Age
[66]	Patients, at risk, & healthy adults from Munich or FePsy database (Retrospective; Germany, Switzerland)	HC: *n* = 437, 32.6 ± 10.9 yrs., 214♀; ARMS: *n* = 89, 24.9 ± 5.8 yrs., 33♀; SZ: *n* = 141, 28.5 ± 7.3 yrs., 33♀; MDD: *n* = 104, 42.3 ± 12.0 yrs., 52♀; BPD: *n* = 57, 25.6 ± 6.7 yrs., 57♀	MRI (T1[1.5 T])	Voxel-wise GM volume & density	SVR [Repeated (×10) nested 10-fold]	SZ, MDD, BPD, & ARMS; SZ disease stage & clinical factors	SZ (5.5 yrs), MD (4.0 yrs), BPD (3.1 yrs), & ARMS (1.7 yrs) GAP ↑ than HC (all *p <* 0.05). ↓ age of onset for MDs (*r* = − 0.26) & BPDs (*r* = − 0.34; both *p* < 0.002). RE- (6.4 yrs), RO-SZ (4.2 yrs), & L-ARMS (2.7 yrs) GAP ↑ than E-ARMS (all *p* < 0.05). ↑ severity in SZ (r ~ 0.20 to 0.26), BPD (r ~ 0.37 to 0.47), & RO-SZ (r ~ 0.27 to 0.30; all *p* < 0.05)	None
[49]	Cases from in- or out-patient services, & community dwelling controls (Germany)	SZ: *n* = 45, 33.7 ± 10.5 (21.4–64.9) yrs., 16♀; BD: *n* = 22, 37.7 ± 10.7 (23.8–57.7) yrs., 12♀; HC: *n* = 70, 33.8 ± 9.4 (21.7–57.8) yrs., 30♀	MRI (T1[1.5 T])	Voxel-wise GM volume	RVR [Data splitting]	SZ & BD	SZ brainAGE (2.56 yrs) ↑ than BD (− 1.25 yrs) & HC (− 0.22 yrs.; both *p* = 0.01). BD comparable to HC (NS). SZ♂(3.37 yrs) ↑ than SZ♀(1.07 yrs.; no *p*-value)	^b^Gender
[30]	1&2) Utrecht Schizophrenia Project, First-Episode Schizophrenia Research Program or GROUP (Longitudinal); 4) Cases & controls from exercise based RCT (both Netherlands)	1&2) SZ: *n* = 341, 29.5 ± 10.0 yrs.; HC: *n* = 386, 34.1 ± 11.8 yrs., at b/line (1-13 yrs. FU); 4) HC: *n* = 55; SZ: *n* = 60, 19-48 yrs.; Sex unknown	MRI (T1[1.5/3 T])	Voxel-wise GM density	SVR [Nested-LOO]	SZ & clinical factors	1&2) SZ GAP + 3.08 yrs. at b/line; ↑ at FU (change = 1.24 yrs.; ^c^rate = 1.36 yrs). Associated with severity & antipsychotic dose at FU (**both** ***p*** **< 0.0025**). Five yrs. post onset, ^c^rate ↓ from 2.5 to 1 yr (no p-value). Associated with severity, no. & duration of hospitalisations, & cumulative antipsychotics (**all** ***p <*** **0.0025**) 4) SZ GAP + 5.59 yrs	None
[34]	Two independent samples of outpatients, & healthy adults from CAMH (Canada)	1) HC: *n* = 41; SSD: *n* = 81, 20–83 yrs.; BD: *n* = 53, 18-81 yrs.; 2) HCs: *n* = 30; SZ: *n* = 67, 40.6 ± 16.3 yrs.; Sex unknown	MRI (T1&DWI[1.5/3 T])	CT, FA, & with (1) or without (2) cognitive scores	RF	SDD & BD	1) SSD GAP (7.8 yrs) ↑ than HCs (0.67 yrs) & BDs (0.14 yrs.; both *p* = 0.001). BDs comparable to HCs (no p-value). 2) SZ GAP (6.12 yrs) ↑ than HC (1.8 yrs., *p* = 0.005)	None
[50]	BD cases from registry, Mood Disorders Program, or University treatment clinic; Controls recruited via advertisement (Canada)	Li: *n* = 41, 47.0 ± 13.8 (20.1–72.3) yrs., 23♀; Non-Li: *n* = 43, 48.2 ± 11.5 (26.9–74.4) yrs., 26♀; HC: *n* = 45, 42.3 ± 13.8 (20.8–70.9) yrs., 21♀	MRI (T1[1.5 T])	Voxel-wise WB volume	RVR [k-fold]	BD with/without Lithium; treatment response (Aldas < 7)	Non-Li brainAGE 4.10 & 4.96 yrs. ↑ than Li & HC, respectively (both *p* < 0.01). Li comparable to HC (NS). Li with partial prophylactic response ↓ than non-Li (*p* = 0.03)	^b^Age

Bold = Results corrected for multiple comparisons; ^a^Brain age adjustment; ^b^Model adjustment; ^c^Calculated by dividing the change in brain age by the time interval between imaging acquisitions; *ADHD* Attention-Deficit/Hyperactivity Disorder; *AlcD* Alcohol dependent patients; *ARMS* At-risk mental states for psychosis; *ASD* Autism spectrum disorder; *AUD* Alcohol Use Disorder; *BD* Bipolar Disorder; *BEA* Brain estimated age; *B/line* Baseline; *BPD* Borderline personality disorder; *CA* Chronological age; *CAD* Cannabis Use Disorder; *CAMH* Centre for Addiction and Mental Health; *CNN* Convolutional Neural Networks; *CT* Cortical thickness; *E-ARMS* Early at-risk mental states for psychosis; *FA* Fractional anisotropy; *FEP* First-episode psychosis; *FePsych* = Früherkennung von Psychosen database; *FU* Follow-up; *GAD* Generalised Anxiety Disorder; *GM* Grey matter; *GROUP* Genetic Risk and Outcome of Psychosis; *HC* Healthy controls; *HR* High risk; *ID* Intellectual disability; *L-ARMS* Late at-risk mental states for psychosis; *LeAD* Learning and Alcohol Dependence; *LC* Lifetime alcohol consumption; *LI* Bipolar Disorder with Lithium treatment; *LOO* Leave one out; *LR* Linear regression; *MDD* Major depression; *MNI* Montreal Neurological Institute registered image; *MRI* Magnetic resonance imaging; *MRR* Multilinear ridge regression; *Non-Li* Bipolar Disorder without Lithium treatment; *NS* Not significant; *ORBIS* Offspring Risk for Bipolar disorders Imaging Study; *RCT* Randomised controlled trial; *RE-ARMS* Recurrently ill at-risk mental states for psychosis; *RO-ARMS* Recent onset at-risk mental states for psychosis; *RR* Ridge regression; *RVR* Relevance vector regression; *SES* Socioeconomic status; *SPECT* Single-photon emission computerized tomography; *SSD* Schizophrenia Spectrum Disorder; *SZ* Schizophrenia; *SVR* Support vector regression; *TICV* Total intracranial volume; *WB* Whole brain; *WM* White matter; *99mTc-HMPAO* Technetium-99 m hexamethylpropylene amine oxime

Fewer studies investigated other psychiatric disorders. There were four studies involving patients with major depression (MD), but with mixed findings. Specifically, two found accelerated brain ageing in MDs, that was significantly different to controls [43, 66]; a second study, involving fewer cases, found no difference between MDs and controls [44], and a third reported decelerated brain ageing but made no statistical comparison to a control group [27]. A fifth study analysed associations in a relatively large sample of community dwelling middle-aged adults, and reported a positive correlation between depression scores and brain ageing [60].

#### Neurological disease

A total of 18 studies investigated brain ageing in relation to neurological diseases, the most common being mild cognitive impairment (MCI), Alzheimer’s Disease (AD) and epilepsy (Table 2). Four of the five studies included a small group of AD participants (ranging between 27 to 76 in size), and reported a significantly higher accelerated brain ageing (ranging between 5.36 and 10 years, at baseline) relative to healthy controls – three sampled participants from the ADNI [33, 55, 56]. The fifth study observed decelerated brain ageing, but using a larger sample of participants with dementia (including AD), and did not statistically compare these findings to a healthy control group [27]. Two studies also included prospective data from the ADNI study, and reported a significantly higher accelerated brain ageing at follow-up, and a greater rate of brain ageing in ADs, relative to healthy controls or participants with stable MCI [33, 56]. All measures of brain ageing (baseline, follow-up and the rate) were significantly higher when participants progressed from MCI to AD, relative to stable MCI and healthy controls [33, 56]. An additional study that also sampled participants from the ADNI study reported a significantly higher accelerated brain ageing (i.e., measured at baseline only) in participants progressing from MCIs onto AD sooner than later, relative to individuals with a stable MCI, or had progressed onto AD at a later stage [58].

**Table 2:** Studies investigating the association between neurological disease and brain ageing

Reference	Study (Design, country)	n, Mean age ± SD (Range), Sex, Other information	Modality (Protocol)	Features	Model (Cross-validation)	Exposure	Main findings outcome	Adjustments
[27]	Multisite psychiatric database (Retrospective; US)	Dementia: *n* = 1622, 54.3 ± 20.7 yrs.; TBI: *n* = 8472, 35.3 ± 15.1 yrs.; Sex unknown	SPECT (rs-99mTc-HMPAO)	Regional cerebral perfusion	LR	Dementia & TBI	CA-BEA ↓ of 4.1 yrs. & 0.19 yrs. in Dementia, & TBI, respectively	None
[68]	J-ADNI study (Japan)	HC: *n* = 146, 68.3 ± 5.6 yrs., 78♀; sMCI: *n* = 102 73.4 ± 6.0 yrs., 57♀; pMCI: *n* = 112, 73.6 ± 5.6 yrs., 65♀; AD: *n* = 147, 74.1 ± 6.6 yrs., 84♀	MRI (T1[1.5 T])	Voxel-wise GM volume	SVR [5-fold]	MCI & AD; cognitive scores; MRI	AD (5.36 yrs), pMCI (3.15 yrs) & sMCI (2.38 yrs) GAP ↑ than HC (0.07 yrs., all *p* < 0.05). Correlates with cognitive scores & MRI (r ~ 0.24–0.28; all *p* < 0.001). WM (NS)	None
[33]	Pooled sample of APOE e4 carriers & non-carriers from ADNI study (Longitudinal; US & Canada)	HC: *n* = 107, 75.7 ± 8.2 yrs.; sMCI: *n* = 36, 77.0 ± 4.1 yrs.; pMCI: *n* = 112, 74.5 ± 7.9 yrs.; AD: *n* = 150, 74.6 ± 9.1 yrs.; 595-1197 days FU; Sex unknown	MRI (T1[1.5 T])	Voxel-wise GM volume	RVR	MCI & AD; cognitive scores	AD & pMCI brainAGE ↑ than sMCI & HC at b/line & FU (**both** ***p*** **< 0.05**). ^d^Rate ↑ in pMCI (0.61–1.13 yrs) & ADs (0.90–1.68 yrs) than sMCI & HC (***p <*** **0.05**). Correlates with cognition at b/line & FU (MMSE: r ~ − 0.34 to − 0.59; ADAS & CDR-SB: r ~ 0.29 to 0.58; all *p* < 0.001)	^b^Age, gender, APOE e4
[55]	ADNI study (US & Canada)	AD: *n* = 102, 75.9 ± 8.3 (55–88) yrs., 55♀; HC: *n* = 232, 76.0 ± 5.1 (60–90) yrs., 113♀	MRI (T1[N/S])	Voxel-wise GM volume	RVR [Data splitting]	Early AD	Early AD brainAGE 10 yrs. ↑ than HC (*p <* 0.001)	None
[56]	ADNI study (Longitudinal; US & Canada)	HC: *n* = 108, 75.6 ± 5.0 yrs., 47♀; sMCI: *n* = 36, 77.0 ± 6.1 yrs., 6♀; pMCI: *n* = 112, 74.5 ± 7.4 yrs., 45♀; AD: *n* = 150, 74.6 ± 7.6 yrs., 74♀; ~ 4 yrs. FU	MRI (T1[1.5 T])	Voxel-wise WB volume	RVR	MCI, & AD; cognitive scores	pMCI & ADs brainAGE ↑ than HC & sMCI at b/line & FU (**both** ***p*** **< 0.05**). Strongest correlation with severity in AD (MMSE: *r* = − 0.46) & cognition in pMCI (ADAS: *r* = 0.40; both *p <* 0.001). ^d^Rate ↑ in pMCI (1.05 yrs) & ADs (1.51 yrs) than sMCI & HC (***p*** **< 0.05**)	^a^Scanner, age, gender
[58]	ADNI study (Longitudinal; US & Canada)	sMCI: *n* = 62, 76.4 ± 6.2 (58–88) yrs., 13♀; Early-pMCI: *n* = 58, 73.9 ± 7.0 (55–86) yrs., 25♀; Late-pMCI: *n* = 75, 75.2 ± 7.3 (56–88) yrs., 27♀ at b/line; 3 yr FU	MRI (T1[1.5 T])	Voxel-wise GM volume	RVR [Data splitting]	sMCI, early & late pMCI; cognitive scores	sMCI (0.75 yrs), early (8.73 yrs) & late pMCI (5.62 yrs) brainAGE (*p <* 0.001). ↑ ADAS & CDR at b/line (r ~ 0.20 to 0.23), that ↑ at FU (r ~ 0.24 to 0.48; all *p* < 0.01). ↓ MMSE at FU only (r ~ − 0.17 to − 0.41; all *p <* 0.05)	None
[67]	The Leipzig Research Centre for Civilization Diseases-Adult-Study (Germany)	OCI-norm: *n* = 729, 59.2 ± 15.2 yrs., 364♀; −mild: *n* = 632, 58.0 ± 14.9 yrs., 294♀; −major: *n* = 251, 58.3 ± 15.7 yrs., 115♀	MRI (T1 & T2*-rs-fMRI [3 T])	Functional, CT, SA, global & subcortical volume	RF stacking (SVR [5-fold])	Normal, mild & major OCI	For all models but stacked-function (NS), OCI-major BA score ↑ (1.52 to 8.68 yrs) than -mild (0.74 to 2.82 yrs), & -norm (− 0.52 to 1.32 yrs.; all *p <* 0.05)	None
[45]	MTLE cases & controls from the Department of Neurology at NTUH (Taiwan)	Right-MTLE: *n* = 17, 37.9 ± 8.1 yrs., 8♀; Left-MTLE: *n* = 18, 37.4 ± 8.5 yrs., 8♀; HC: *n* = 37, 38.4 ± 8.3 yrs., 20♀	MRI (DSI [3 T])	Compact features of 7 diffusion indices & 76 fibre tract bundles	GPR [10-fold]	R & L MTLE; clinical characteristics	R-MTLE GAP (10.94 yrs) ↑ than L-MTLE (2.24 yrs) & HCs (0.82 yrs.; **both** ***p <*** **0.05**). L-MTLE comparable to HC (NS). Correlates with age of onset (R: *r* = − 0.51; L: ρ = 0.59; both *p <* 0.05), & illness duration (R: *r* = 0.50, *p* = 0.040; L: *r* = − 0.48, *p* = 0.049). ↑ seizure frequency for R-MTLE only (ρ = 0.64, *p* = 0.007)	^b^Age, gender, no. of AED classes, handedness
[69]	Patients from ECP; & healthy adults from ECP or ADCP (US)	TLE: *n* = 104, 40.4 ± 11.8 (19–60) yrs., 64♀; HC: *n* = 151, 53.7 ± 19.4 (18–89) yrs., 88♀	MRI (T1 & rs-fMRI[3 T])	CT, SA & volume/rs-correlation matrices	SVR [10-fold]	TLE; clinical characteristics & AEDs	TLE structural & functional GAP 6.6 & 8.3 yrs., respectively. Functional correlates ↑ complex partial seizures (ρ = 0.300) & no. of AEDs (ρ = 0.279; **both** ***p*** **= 0.07**)	^a^Age
[40]	Cases from New York University treatment center, or HEP; community dwelling controls (US, AUS & Canada)	MRE: *n* = 94, 32.3 ± 13.6 yrs., 46♀; NDE: *n* = 42, 31.4 ± 11.4 yrs., 21♀; Matched HC: *n* = 74, 28.9 ± 10.2 yrs., 41♀	MRI (T1[3T])	Voxel-wise WB volume	GPR [10-fold]	MRE & NDE; clinical factors	MREs brainAGE 4.5 yrs. ↑ than HC (0 yrs., *p* = 4.6 × 10–5). NDE comparable to HC (NS). NS with duration. BrainAGE ↓ with ↑ age of MRE onset (− 0.15 yr per year, *p* = 0.03).	^b^Age, gender
[52]	Epileptic or PNES cases, & healthy controls from authors institute (Location unknown)	TLE-NL: *n* = 164, 45.8 ± 16.6 yrs., 83♀; TLE-HS: *n* = 63, 43.3 ± 13.7 yrs., 38♀; Ext-FE: *n* = 45, 35.9 ± 12.0 yrs., 18♀; IGE: *n* = 30, 28.9 ± 7.7 yrs., 22♀; PME/SGE: *n* = 5, 31.4 ± 9.8 yrs., 2♀; PNES: *n* = 11, 31.5 ± 8.6 yrs., 8♀	MRI (T1[3T])	Voxel-wise WB volume	SVR [10-fold]	Epilepsy; TLE with/without psychosis; PNES/ MRI(−); PME/JME	GAP ↑ in epileptics than HCs (~ 4.7 to 21.2 yrs., *p <* 0.01), exception being Ext-FE (NS). ↑ for TLEs with psychosis (10.9 yrs) than without (5.3 yrs.; *p <* 0.001). PNES comparable to MRI(−) (NS). PME (22.0 yrs) ↑ than JME (9.3 yrs.; no p-value)	^b^Age, gender
[51]	Cases with persistent neurological problems following TBI, & healthy controls (Location unknown)	TBI: *n* = 99, 38.0 ± 12.4 yrs., 27♀; HC: *n* = 113, 43.3 ± 20.2 yrs., 64♀	MRI (T1[3T])	Voxel-wise GM or WM volume	GPR [10-fold (×1000)]	TBI; cognitive function; TSI	TBI PAD ↑ than HC (both *p <* 0.01). ↑ TSI (GM & WM: r ~ 0.50 to 0.54; both *p <* 0.001). In TBI, ↑ processing speed (ρ ~ 0.27 to 0.38) & ↓ recall (ρ = − 0.25; all *p <* 0.05). GM PAD correlates executive function (ρ ~ 0.25 to 0.27; all *p <* 0.05)	^b^Age, gender
[70]	Service members from Iowa City Veterans Affairs Medical Center (Retrospective; US)	With TBI: *n* = 92, 29.7 ± 7.0 (22–57) yrs., 4♀; Without TBI: *n* = 34, 31.1 ± 9.2 (22–55) yrs., 4♀	MRI (T1[3T])	CT	LR, RF, SVR & GPR [Random half-split (× 10,000)]	TBI & characteristics	TBI GAP ↑ than HCs (all models but RF, *p <* 0.05). TBI characteristics NS	None
[28]	Patients scanned at MAGNISM center, or Imperial College London, & healthy controls (Longitudinal; UK, Italy, Austria, Catalonia & Netherlands)	MS & CIS: *n* = 1204, 39.4 ± 10.8 (15–68) yrs., 771♀, 0.2-15 yrs. FU; HC: *n* = 150, 37.3 ± 10.0 (23–66) yrs., 82♀, 0.5–6.0 FU	MRI (T1[1.5/3 T])	Voxel-wise WB volume	GPR [10-fold]	MS & CIS; b/line & annual severity of disability (EDSS)	MS & CIS GAP (10.3 yrs) ↑ than HC (4.3 yrs., *p <* 0.001). ↑ in SPMS (13.3 yrs). CIS comparable to HC (NS). MS & CIS ^c^rate (0.61 yrs) ↑ than HC (− 0.17 yrs., *p* = 0.016); differs between sub-groups (*p* = 0.002). GAP 0.64 yrs. per 1 EDSS (*p <* 0.001). ↑ ^c^Rate with ↑ annual EDSS (*r* = 0.26, *p <* 0.001)	^ab^Age; ^b^Gender, age^2^, scanner, cohort, treatment, normalized brain volume
[47]	MS cases & matched controls from local community, or registry (Case-control & longitudinal; Norway)	MS: *n* = 76, 21–49 yrs., 71%♀. 2 FU approx. 26 & 66mths; HC: *n* = 235, 26-53 yrs., 72%♀	MRI (T1[1.5/3 T])	CT, SA & volume	Xgboost [Nested with 10-fold]	MS; clinical factors; MRI	All but temporal GAP (global & regional: ~ 2.4 to 6.2 yrs) ↑ than HC (all *p <* 0.05). In MS, ↑ atrophy (global & regional: r ~ 0.28 to 0.41), WMLL (global: *r* = 0.46; cereb./subcort: *r* = 0.38) & volume (regional: r ~ − 0.35 to − 0.43; **all** ***p <*** **0.05**). Global ^c^rate 0.41 yrs. (*p* = 0.008). ↑ atrophy & WMLL (**all global and regional,** ***p*** **< 0.05**). GAP & ^c^rate not associated with clinical factors (NS)	^a^Age, age^2^, gender, scanner
[59]	Cognition and Neocortical Volume after Stroke (CANVAS; Prospective, AUS)	Stroke (6wks post event): *n* = 135, 67.4 ± 13.0 yrs., 41♀; HC: *n* = 40, 68.7 ± 6.6 yrs., 15♀; 3 & 12mth FU	MRI (T1[3T])	CT, SA & subcortical volume	Stacked RF (SVR)	Ischemic stroke (6wks, 3 & 12mths after event)	3mths post stroke, BA score was 3.9 to 8.7 yrs. ↑ than HCs (*p <* 0.01). ^d^Rate over 1 yr did not differ between HC & stroke patients (no results provided)	^b^Education (yrs)
[71]	Mild stroke patients attending doubled-blind randomised control trial (Norway)	*n* = 54, 69.7 ± 7.5 (47.8–82.0) yrs., 14♀; 3wk intervention 6mths after admission	MRI (T1[3T])	Global & regional CT, SA & volume	Xgboost [10-fold]	Cognitive function & improvement	**GAP NS following FDR**	^ab^Age, gender; ^a^age^2^
[48]	Cases & healthy controls recruited at hospital, via personnel or local support groups (US)	PD: *n* = 37, 58.8 ± 10.9 yrs., 17♀; HC: *n* = 20, 47.0 ± 17.1 yrs., 10♀	PET (18F-FDG)	CMRGlc & GMR	LR	PD & clinical factors	PDs GAP ↓ than HCs (*p <* 0.005). ↑ duration (*r* = − 0.38, *p* < 0.04), & severity (~*r* = − 0.39 to − 0.32, *p* ≤ 0.05). Mean preclinical period of 4.5 yrs. (no details provided)	^a^Age

Bold = Results corrected for multiple comparisons; ^a^Brain age adjustment; ^b^Model adjustment; ^c^Calculated by dividing the change in brain age by the time interval between imaging acquisitions; ^d^Calculated by regressing time on brain age; *18F-FDG* [18F]fluorodeoxyglucose; *99mTc-HMPAO* Technetium-99 m hexamethylpropylene amine oxime; *AD* Alzheimer’s Disease; *ADAS* Alzheimer’s Disease Assessment Score [72–74]; *ADCP* Alzheimer’s Disease Connectome Project; *ADNI* Alzheimer’s Disease Neuroimaging Initiative; *AED* Anti-epileptic drug; *B/line* Baseline; *CDR/SB* Clinical Dementia Rating/‘sum of boxes’ [75]; *Cereb/subcort* Cerebellar/subcortical features; *CIS* Clinically isolated syndrome; *CMRGlc* Cerebral metabolic rate for glucose; *CSF* Cerebral spinal fluid; *CT* Cortical thickness; *DSI* Diffusion spectrum imaging; *ECP* Epilepsy Connectome Project; *EDSS* Expanded Disability Status Scale [76]; Ext-FE = Extra-temporal lobe focal epilepsy; FAQ = Functional Assessment Questionnaire [77]; *FU* Follow-up; *GM* Grey matter; *GMR* Global metabolic rate for glucose; *GPR* Gaussian process regression; *HC* Healthy controls; *HEP* Human Epilepsy Project; *IGE* Idiopathic generalized epilepsy; *J-ADNI* Japan Alzheimer’s Disease Neuroimaging Initiative; *JME* Juvenile myoclonic epilepsy; *LR* Linear regression; *MAGNISM* Magnetic Resonance Imaging in Multiple Sclerosis; *MMSE* Mini-Mental State Examination [78, 79]; *MRE* Medically refractory focal epilepsy; *MRI(−)* Magnetic resonance imaging negative epilepsy; *MRI* Magnetic resonance imaging; *MS* Multiple Sclerosis; *MTLE* Mesial temporal lobe epilepsy; *NDE* Newly diagnosed focal epilepsy; *NS* Not significant; *N/S* Not specified; *NTUH* National Taiwan University Hospital; *OCI* Objective cognitive impairment; *PD* Parkinson’s Disease; *PET* Positron emission tomography; *pMCI* Progressive mild cognitive impairment; *PME* Progressive myoclonus epilepsy; *PNES* Psychogenic nonepileptic seizures; *RF* Random forest; *Rs-fMRI* Resting state functional magnetic resonance imaging; *RVR* Relevance vector regression; *SA* Surface area; *SGE* Symptomatic generalized epilepsy; *sMCI* Stable mild cognitive impairment; *SPECT* Single-photon emission computerized tomography; *SPMS* Secondary-progressive multiple sclerosis; *SVR* Support vector regression; *TBI* Traumatic brain injury; *TLE* Temporal lobe epilepsy; *TLE-NL* Temporal lobe epilepsy with visually normal MRI; *TLE-HS* Temporal lobe epilepsy with hippocampal sclerosis; *TSI* Time since injury; *WB* Whole brain; *WMLL* White matter lesion load; *WM* White matter; *Xgboost* Extreme gradient boosting

Beyond looking specifically at diagnostic categories of dementia, four studies also correlated brain ageing with cognitive scores. These studies used similar cognitive measures (Mini-Mental State Examination (MMSE) [78, 79], Clinical Dementia Rating (CDR)/CDR-sub of boxes [75] or Alzheimer’s Disease Assessment Scale (ADAS) [72–74]) but reported mixed results [33, 56, 58, 68]. Of the three studies including participants from the ADNI, one observed a significant correlation between brain ageing and each of the CDR, ADAS, and MMSE at both baseline and follow up, when pooling healthy controls with diagnostic groups [33]. A second study only included those with MCI, and observed a correlation with CDR and ADAS at baseline that increased at each follow up; correlations with MMSE were observed only at follow up [58]. A third study reported the strongest correlations in individuals with AD was between brain ageing and MMSE, and in progressive MCI with ADAS [56]. When pooling healthy controls with diagnostic groups, an alternative fourth study also observed a correlation with the CDR, ADAS, MMSE, [68].

Four studies investigated brain ageing in relation to various types of epilepsy [40, 45, 52, 69]. Specifically, two studies focused on small groups (ranging between 17 to 104) of participants with temporal lobe epilepsy, and report accelerated brain ageing [45, 69]. However, one was a case-control study that observed a significant difference to healthy controls, but only when seizures were localised to the right hemisphere [45], while the second, slightly larger cohort study had not statistically compared these findings to healthy controls [69]. The two-remaining case-control studies investigated brain ageing in patients with other forms of epilepsy. One compared brain ageing in medical refractory epilepsy (MRE) (~ 50% of the patients experienced seizures in the temporal lobe) to newly diagnosed focal epilepsy (NDE), and reported significant accelerated brain ageing in MREs only, as NDEs were comparable to healthy controls [40]. The second reported accelerated brain ageing in all participants with epilepsy (i.e., focal and generalised), including neuropsychiatric conditions with episodes that resemble epileptic seizures (i.e., psychogenic nonepileptic seizures), except those with extra-temporal lobe focal epilepsy, had a significantly higher accelerated brain ageing than healthy controls [52, 80].

Fewer studies analysed the effects of stroke [59, 71], traumatic brain injury (TBI) [27, 51, 70], multiple sclerosis (MS) [28, 47], or Parkinson’s disease on brain ageing [48]. Three studies analysed brain ageing in TBI patients, but report mixed results. Specifically, two smaller sample studies found significantly higher accelerated brain ageing in TBI patients relative to healthy controls [51, 70]; a third reported decelerated brain ageing for a large cohort of TBI patients, but did not statistically compare findings to other diagnostic groups [27]. The two former studies also investigated time since TBI, but only one found a significant positive correlation with the time since TBI [51, 70].

Of the remaining studies, two reported greater cross-sectional estimates of accelerated brain ageing for patients with MS relative to healthy controls [28, 47]. Longitudinal assessments by one of these two studies resulted in a higher annual rate of accelerated brain ageing in a large pooled sample of MS and clinically isolated syndrome patients (i.e., individuals with a greater likelihood of MS), relative to healthy controls [28, 81]; the second did not compare findings to healthy controls, but also observed an annual accelerated rate of brain ageing when using a much smaller sample of MS patients [47]. In stroke patients, one randomised control study found no correlation between regional or global estimates of brain ageing with cognitive function [71], while a second prospective cohort study found a significantly higher brain ageing than healthy controls, despite features used to estimate brain age [59]. For the latter study, however, the direction of brain ageing (i.e., accelerated/decelerated) varied between models, for both patients and controls [59]. From this study, the rate of brain ageing was also comparable between patients and healthy controls, though no statistics were reported [59].

#### Health, physical and biological markers

Fourteen studies investigated brain ageing in relation to diseases without a primary neurological presentation (Human Immunodeficiency Virus (HIV) and type II diabetes), markers of health (e.g., biological and physical), hormones, medications, chronic pain, or mortality risk (Table 3) [19, 36, 38, 39, 53, 54, 57, 60–63, 66, 82, 83]. Most commonly reported were associations with body mass index (BMI) [38, 53, 57, 66]. Of the four studies investigating BMI, two involved community dwelling, initially healthy older adults from the ADNI cohort study or the UK Biobank, while the other two studies sampled young adult patients with SZ [38, 53, 57, 66]. The two former studies both reported a positive correlation with BMI, however, the larger cohort study observed this association when predicting age for both genders, or females only [53]; while the second, smaller sample study reported this effect in males only when defined ageing for the total sample [53, 57]. A significant positive association with BMI was also reported in SZ patients. However, one study found this effect to be independent to an SZ diagnosis (i.e., main effects of BMI and SZ on brain ageing were evident, but no significant BMI-by-SZ interaction); while the second only observed an association for a smaller group of patients with a recent onset of SZ [38].

**Table 3:** Studies investigating health, physical and biological markers, hormones and medications, and disease

Reference	Study (Design, country)	n, Mean age ± SD (Range), Sex, Other information	Modality (Protocol)	Features	Model (Cross-validation)	Exposure	Main findings outcome	Adjustments
[82]	Co-morbidity in Relation to AIDS (COBRA; UK & Netherlands)	HIV+: *n* = 162, 56 (51–62) yrs., 9♀; HIV-: *n* = 105, 55.8 (50–62) yrs., 6♀	MRI (T1[3T])	Voxel-wise WB volume	GPR [10-fold (×1000)]	HIV; clinical & health factors	HIV+ PAD ↑ than HIV- (b = 3.31, *p <* 0.01). NS with clinical or health factors	^b^Scanner, gender, ICV, smoking
[39]	HIV positive & negative adults from a larger study (K23 MH095661)	HIV+: *n* = 70, 50.7 ± 11.9 (24–76) yrs., 12♀; HIV-: *n* = 34, 53.3 ± 10.3 (24–66) yrs., 17♀	MRI (DWI[3 T])	FA, L1, AD, MD	SVR [10-fold]	HIV; clinical factors; cognitive function	HIV+ GAP ↑ than HIV- (n2 = 0.21, *p* = 0.001). HIV+ associated with viral load (β = 0.23, ***p*** **= 0.03**), & cognitive function (learning: *r* = − 0.26; memory: *r* = − 0.21; **both** ***p*** **= 0.03**)	^a^Scanner; ^b^Age, gender, duration, HAART, CD4, viral load, comorbidity
[62]	Central nervous system HIV Anti-Retroviral Therapy Effects Research (CHARTER; Longitudinal; US)	*n* = 139, median age 44 yrs.(IQR: 44-55 yrs), 19♀; *n* = 111 with FU cognitive data (mean 3.5 visits)	MRI (T1[1.5 T])	Voxel-wise WB volume	GPR	HIV; clinical factors; comorbidity; cognitive function, deficit, & change	GAP + 1.17 yrs. ↑ for HIV+ with confounding comorbidity (5.87 yrs., *p* < 0.01). Trend with prior AIDS (3.03 yrs., *p* = 0.05). ↑ cognitive deficit (b = 0.011, *p* = 0.03). No association with cognitive function, or change in function (NS)	^b^Age, comorbidity, scanner, TICV
[38]	Cases from the Early Stages of Schizophrenia, & community dwelling controls (Czech Republic)	FEP: *n* = 120, 27.0 ± 4.9 (18–35) yrs., 46♀; HC: *n* = 114, 25.7 ± 4.0 (18–35) yrs., 51♀	MRI (T1[3T])	Voxel-wise WB volume	RVR	Obesity, LDLs, HDLs & triglycerides	↑ BrainAGE in obese/overweight (B = 0.92, *p <* 0.01). ↑ in obese/overweight FEPs (3.83 yrs., 95% CI: 2.35–5.31 yrs); ↓ in normal weighted HC (− 0.27 yrs., 95% CI: − 1.22-0.69 yrs). LDL, HDL, & triglycerides NS	^b^Age
[66]	Patients, at risk, & healthy adults from Munich or FePsy database (Retrospective; Germany, Switzerland)	HC: *n* = 437 32.6 ± 10.9 yrs., 214♀; ARMS: *n* = 89, 24.9 ± 5.8 yrs., 33♀; SZ: *n* = 141, 28.5 ± 7.3 yrs., 33♀; MDD: *n* = 104, 42.3 ± 12.0 yrs., 52♀; BPD: *n* = 57, 25.6 ± 6.7 yrs., 57♀	MRI (T1[1.5 T])	Voxel-wise GM volume & density	SVR [Repeated (×10) nested 10-fold]	BMI	GAP correlates with ↑ BMI in RO-SZ only (*r* = 0.36, *p <* 0.05)	None
[53]	UK Biobank	*n* = 19,000, 10,112♀; Age unknown	MRI (T1, rfMRI, tfMRI, T2 FLAIR, dMRI, swMRI [3 T])	IDP	Non-LR [10-fold]	^d^Body composition; bone density; blood pressure, heart rate; haemoglobin; health & medications	GAP correlates with body composition, & bone density (r ~ − 0.08 to − 0.18); also observed in ♀ (all -log_10_P > 8). Correlations with no. treatments/medications, & diabetes (both *r* = 0.06); also observed in ♂ (all -log_10_P > 8). Strongest correlation with blood pressure, heart rate, & haemoglobin in ♂ (r ~ 0.08 to 0.11; all -log_10_P > 8)	^a^Age, age^2^, gender
[19]	Lothian Birth Cohort 1936 (LBC1936; Scotland)	*n* = 669 (*n* = 73 deceased), 72.7 ± 0.7 yrs., 317♀	MRI (T1[3T])	Voxel-wise WB volume	GPR [10-fold (× 1000)]	Mortality	Deceased ♂ & ♀ PAD 8.13 & 2.07 yrs., respectively. Surviving ♂ & ♀ PAD 3.76 & -1.64 yrs., respectively	None
[54]	UK Biobank	*n* = 14,701, 62.6 ± 7.5 yrs., 7914♀	MRI (T1, rfMRI, tfMRI, T2 FLAIR, dMRI, swMRI [3 T])	IDP	LASSO [10-fold]	Blood pressure; diabetes & stroke history	↑ GAP associated with health (DBP: B = 0.05; SBP: B = 0.03; diabetes: B = 2.12; stroke history: B = 2.70; **all** ***p*** **< 0.001**)	^ab^Age; ^b^Age^2^, gender, height, volumetric scaling, & tfMRI head motion
[60]	Community dwelling adults from 1 of 6 studies (Longitudinal; US)	2DM: *n* = 98, 64.6 ± 8.1 yrs., 45♀; HC: *n* = 87, 65.3 ± 8.5 yrs., 46♀, at b/line; 3.8 ± 1.5 yrs. (*n* = 25) FU	MRI (T1[3T])	Voxel-wise WB volume	RVR	2DM; clinical laboratory data	2DM brainAGE (4.6 yrs) ↑ than HC (*p <* 0.0001). ^c^Rate ↑ by 0.2 yrs. per FU (*p* = 0.03). In total cohort, ↑ TNFa (*r* = 0.29, *p* = 0.01). 2DM correlates hyperglycemia (*r* = 0.34) & duration (*r* = 0.31; both *p* < 0.05)	^b^Age, gender, hypertension, diabetes duration
[57]	Males & females from ADNI (US & Canada)	*n* = 118♂, 75.8 ± 5.3 (60–88)yrs.; *n* = 110♀, 76.1 ± 4.8 (62–90)yrs	MRI (T1[1.5 T])	Voxel-wise GM volume	RVR	Physiological & clinical chemistry markers	♂ brainAGE correlates ↑ BMI, DBP, GGT, & uric acid (r ~ 0.19 to 0.35; all *p <* 0.05). ♀ correlates ↑ GGT, AST, ALT (r ~ 0.20 to 0.25), & ↓ B12 (*r* = − 0.17; all *p <* 0.05)	^b^Gender, age, site
[83]	Neuromodulatory Examination of Pain and Mobility Across the Lifespan (NEPAL; US)	NCP: *n* = 14, 71.5 ± 7.3 yrs., 8♀; CP: *n* = 33, 70.6 ± 5.5 yrs., 27♀	MRI (T1[3T])	Voxel-wise WB volume	GPR [10-fold]	CP; pain characteristics; psychological & emotional function	CPs PAD (1.5 yrs) ↑ than NCPs (− 4.0 yrs., *p* = 0.03). Correlates with positive affect (ρ = − 0.47, ***p*** **= 0.04**) & average intensity of worst pain (*r* = 0.46, *p* = **0.03**)	^b^Age, exercise, gender
[36]	Young postpartum women (Longitudinal; Sweden)	Early-: *n* = 14, 32.8 ± 4.0 (25–38)yrs.; Late-postpartum: 35 ± 5 days later	MRI (T1[3T])	Voxel-wise GM volume	RVR	Early & late post-partum; estradiol & progesterone	Late postpartum BrainAGE 5.36 yrs. ↓ than early (*p <* 0.001). No correlation with estradiol, or progesterone (data not provided)	None
[61]	Female volunteers with known ovulation cycle, & paired males (Longitudinal)	7♀, 21–31 yrs.; 7♂, 23-37 yrs. at t1; Scanned at ovulation (t2), midluteal phase (t3) & next menses (t4)	MRI (T1[1.5 T])	Voxel-wise GM volume	RVR	Menstrual cycle; estradiol & progesterone	BrainAGE differs during cycle (*p* = 0.03). ↓ From t1–2 (1.27 yrs., *p <* 0.05); NS from t1–3 (0.5 yrs) & from t1–4 (0.10 yrs). Correlates with estradiol only (*r* = − 0.42, *p <* 0.05)	None
[63]	Community dwelling adults from double-blinded randomised control trial (US)	*n* = 20, 32.4 ± 6.7 (23–47) yrs., 10♀; 2 two week FU	MRI (T1[3T])	Voxel-wise GM volume	SVR [10-fold]	Acute Ibuprofen before scan (200 & 600 mg)	Ibuprofen associated with ↓ GAP (200 mg: β = −1.18 yrs., *p* = 0.005; 600 mg: β = − 1.15 yrs., *p* = 0.006)	None

Bold = Results corrected for multiple comparisons; ^a^Brain age adjustment; ^b^Model adjustment; ^c^Calculated by regressing time on brain age; ^d^Body composition = Body mass index (*BMI*), weight, hip circumference, right arm fat mass, body fat percentage, abdominal subcutaneous adipose tissue volume; Bone density = Heel bone mineral density (BMD), total BMD, total bone mineral content and head BMD; Haemoglobin = Mean corpuscular haemoglobin, mean corpuscular volume; Blood pressure = Systolic and diastolic blood pressure. *AD* Axial diffusivity; *ADNI* Alzheimer’s Disease Neuroimaging Initiative; *ALT* Albumin alanin-aminotransferase; *ARMS* At-risk mental states for psychosis; *AST* Aspartate-aminotransferase; *B12* Vitamin B12; *B/line* Baseline; *BPD* Borderline personality disorder; *DBP* Diastolic blood pressure; *2DM* Type 2 diabetes mellitus; *dMRI* Diffusion magnetic resonance imaging; *DWI* Diffusion weighted imaging; *FA* Fractional anisotropy; *FLAIR* T2-weighted fluid-attenuated inversion recovery structural imaging; *FEP* First-episode psychosis; *FePsych* Früherkennung von Psychosen; *FU* Follow-up; *GGT* γ-glutamyltransferase; *GM* Grey matter; *GPR* Gaussian process regression; *HAART* Highly active anti-retroviral therapy; *HC* Healthy controls; *HDL* High density lipoproteins; *HIV+/−* Human Immunodeficiency Virus positive or negative; *IDP* Imaging derived phenotypes (i.e., summary measures of structural and functional brain phenotypes); *L1* Radial diffusivity; *LASSO* Least absolute shrinkage and selection operator regression; *LDL* Low density lipoproteins; *LR* Linear regression; *MDD* Major depression; *MD* Mean diffusivity; *MRI* Magnetic resonance imaging; *N/CP* With or without chronic pain; *NS* Not significant; *rfMRI* Resting state functional magnetic resonance imaging; *RO-ARMS* Recent onset at-risk mental states for psychosis; *RVR* Relevance vector regression; *SBP* Systolic blood pressure; *SVR* Support vector regression; *swMRI* Susceptibility-weighted imaging; *SZ* Schizophrenia; *tfMRI* Task functional magnetic resonance imaging; *T/ICV* Total/Intracranial volume; *TNFa* Tumor necrosis factor alpha; *WB* Whole brain

Three small cohort studies (≤162 participants) analysed the effects of HIV [39, 62, 82]. Regardless of model and feature type, all studies reported accelerated brain ageing in HIV positive patients (ranging between 1.17 and 5.87 years). For two studies, this brain ageing was significantly higher than HIV-negative controls [39, 82]; while a third study’s findings were relative only to the model (i.e., a null hypothesis that predicted minus chronological age equals zero) [62]. Associations between brain ageing and HIV clinical characteristics (e.g., years since diagnosis, cell counts (CD4)) were also investigated. One study reported an association between higher brain ageing and prior Acquired Immuno-Deficiency Syndrome status [62] whilst another with viral loading [39]. In contrast, a third observed no significant association with any of the clinical or health factors (all *p* > 0.10) [82].

Two studies considered the influence of female sex hormones, however, one in the context of pregnancy, while the other during a normal menstrual cycle [36, 61]. Both studies relied on small sample sizes of young adult women (≤14 participants). Neither study found significant correlations between brain ageing and progesterone [36, 61] but one reported a significant negative correlation with estradiol (i.e., measured at time point two, when it was most elevated) [61].

#### Environmental and lifestyle factors

Seven eligible studies investigated environmental influences on brain ageing with the most common being smoking and alcohol consumption (Table 4) [53, 54, 60]. Two of the three studies involved a large sample of participants from the UK Biobank, and report a positive association between brain ageing (estimated using different algorithms) and alcohol intake, however, the second also observed a correlation when estimating brain age for females only [53, 54]. Both studies also reported a significant positive correlation with smoking [53, 54]. A third independent study also reported a significant, positive association with smoking, and alcohol, but for fewer community dwelling adults [60]. Meditation practitioners, and amateur/professional musicians were reported to have a significantly lower brain ageing than controls, but were each analysed by one study [29, 42]. Similarly, one study found a higher education, or a greater flight of stairs climbed, to be significantly associated with decelerated brain ageing [64].

**Table 4:** Studies investigating the association between positive and negative environmental and lifestyle factors

Reference	Study (Design, country)	n, Mean age ± SD (Range), Sex, Other information	Modality (Protocol)	Features	Model (Cross-validation)	Exposure	Main findings	Adjustments
[35]	Military serving male twin pairs from VETSA MRI cohort (US)	*n* = 359, 61.8 ± 2.6 (56.5–65.6)yrs.; ~ 5 yr FU	MRI (T1[3T])	CT, SA & subcortical volume	SVR	Negative midlife FLE	GAP + 2.3 yrs. (~ −21.1 to 14.8 yrs). ↑ Total FLE (β = 0.14, *p* = 0.01). Minus extreme outliers, ↑ relationship FLEs (β = 0.11, *p* = 0.03). NS with financial (β = 0.06) or health FLEs (β = 0.05)	^b^Age, scanner, relatedness, cardiovascular risk, alcohol, SES, ethnicity
[60]	Community dwelling adults from 1 of 6 studies (US)	*n* = 185, 64.9 ± 8.3 yrs., 91♀	MRI (T1[3T])	Voxel-wise WB volume	RVR	Smoking & alcohol	↑ BrainAGE with smoking (*r* = 0.20, *p* = 0.008) & alcohol (*r* = 0.24, *p* = 0.001)	^b^Age, gender, diabetes duration
[53]	UK Biobank	*n* = 19,000, 10,112♀; Age unknown	MRI (T1, rfMRI, tfMRI, T2 FLAIR, dMRI, swMRI [3 T])	IDP	Non-LR [10-fold]	Smoking; alcohol; time outdoors; ^c^SES	GAP correlated with smoking (r ~ 0.07 to 0.08; all -log10P > 8). Correlation with alcohol also observed in ♀ (both *r* = 0.07; −log_10_P > 8). Correlation with SES also observed in ♂ (r ~ − 0.05 to − 0.04; all -log10P > 8)	^a^Age, age^2^, gender
[54]	UK Biobank	*n* = 14,701, 62.6 ± 7.5 yrs., 7914♀	MRI (T1, rfMRI, tfMRI, T2 FLAIR, dMRI, swMRI [3 T])	IDP	LASSO [10-fold]	Smoking & alcohol	GAP associated with smoking (B = 0.879) & alcohol (B = -0.997; **both** ***p*** **< 0.001**)	^ab^Age; ^b^age^2^, gender, height, volumetric scaling, & tfMRI head motion
[42]	Meditation practitioners from greater Los Angeles; matched controls from ICBM (US)	Meditators: *n* = 50, 51.4 ± 12.8 yrs., 22♀; HC: *n* = 50, 51.4 ± 11.8 yrs., 22♀	MRI (T1[1.5 T])	Voxel-wise GM volume	RVR	Meditation	BrainAGE associated with meditation (β = −7.53, *p* = 0.047). For every yr > 50 yrs., meditators were 1mth & 22 days younger (β = − 0.14, *p* = 0.045)	^b^Age, gender, handedness, group
[64]	Manhattan or New Jersey community dwelling adults attending of 1 of 3 independent studies (US)	*n* = 331, 19-79 yrs., 182♀	MRI (T1[3T])	Cortical & subcortical GM volume	SSM [Bootstrapping (×1000)]	Education & physical activity (FOSC)	CA-BA associated with ↑ education (β = 0.95), & FOSC (β = 0.58; both *p* = 0.005)	^a^TICV, study, gender; ^b^education, different exercises
[29]	Adults differing in musician status (Case-control, location unclear)	Professionals: *n* = 42, 24.3 ± 3.9 (18–39) yrs., 22♀; Amateurs: *n* = 45, 24.3 ± 3.9 (17–34) yrs., 18♀; Non-musicians: *n* = 38, 25.2 ± 4.8 (17–39) yrs., 15♀	MRI (T1[1.5 T])	Voxel-wise GM volume	RVR	Musician status & years of music	Musicians brainAGE 4.12 yrs. ↓ than non-musicians (*p* = 0.004). Professionals (−3.70 yrs) ↓ than non-musicians (− 0.48 yrs., ***p*** **= 0.014**). Amateurs comparable to non-musicians (**NS**). ↑ music making for professionals only (*r* = 0.32, *p* = 0.04)	^a^Non-musician median brainAGE

Bold = Results corrected for multiple comparisons; ^a^Brain age adjustment; ^b^Model adjustment; ^c^SES = Includes measures of average total household income before tax & number in household. *CT* Cortical thickness; *dMRI* Diffusion magnetic resonance imaging; *FLAIR* T2-weighted fluid-attenuated inversion recovery structural imaging; *FLE* Fateful life events; *FU* Follow-up; *GM* Grey matter; *HC* Healthy controls; *ICBM* International Consortium for Brain Mapping; *IDP* Imaging derived phenotypes (i.e., summary measures of structural and functional brain phenotypes); *LASSO* Least absolute shrinkage and selection operator regression; *LR* Linear regression; *MRI* Magnetic resonance imaging; *NS* Not significant; *RfMRI* Resting state functional magnetic resonance imaging; *RVR* Relevance vector regression; *SA* Surface area; *SES* Socio-economic status; *SSM* Scaled subprofile modelling; *SVR* Support vector regression; *swMRI* Susceptibility-weighted imaging; *tfMRI* Task functional magnetic resonance imaging; *TICV* Total intracranial volume; *VETSA MRI* Vietnam Era twin study of ageing; *WB* Whole brain

#### Genetic influences

Five studies investigated genetic influences on brain ageing (Table 5). Two studies reported no significant difference in brain ageing due to Apolipoprotein E (APOE) e4 carrier status in older adults [33, 84]. One, however, used prospective data from the ADNI study, and found a significantly higher rate of accelerated ageing in APOE e4 carriers [33]. Both study samples, however, involved a limited number of participants (≤101 participants), and thus may be under-powered.

**Table 5:** Studies investigating the association between genetics and brain ageing

Reference	Study (Design, country)	n, Mean age ± SD (Range), Sex, Other information	Modality (Protocol)	Features	Model (Cross-validation)	Exposure	Main findings outcome	Adjustments
[26]	1) Cases recruited from University; controls from NSPN U-Change, or local population; 2) SNORD116 case; controls from 1 of 6 studies (both UK)	1) PWS: *n* = 20, 23.1 ± 2.4 (19–27) yrs., 6♀; HC: *n* = 40, 22.9 ± 2.2 (19–29) yrs., 14♀; 2) 1 ♂, 24.5 yrs.; Matched HC: *n* = 95, 34.0 ± 10.2 (19.9–55.5) yrs., 58♀	MRI (T1[3T])	Voxel-wise WB volume	GPR [10-fold (×1000)]	PWS; SNORD116; clinical characteristics	1) PWS PAD ↑ than HC (7.24 yrs), even when matched for BMI (5.51 yrs.; both *p* < 0.05). No association with PWS IQ, growth or sex hormones, medications, & behaviour (NS); 2) SNORE116 ↑ than HC (12.03 yrs., no *p*-value)	^b^BMI, group differences
[46]	Case-control study on DS (England & Scotland)	DS: *n* = 46, 42.3 ± 9.8 (28–65) yrs., 41♀; HC: *n* = 30, 46.2 ± 9.8 (30–64) yrs., 14♀	MRI (T1[1.5 T])	Voxel-wise WB volume	GPR [10-fold (×1000)]	DS; cognitive status; PiB uptake	DS PAD ↑ than HC (b = 7.69, *p* < 0.001). PiB+ DS (*n* = 19) 5.29 yrs.; PiB- (*n* = 27) 0.52 yrs. Cognitive subgroups (i.e., stable, declining/dementia) comparable (NS)	None
[84]	Community dwelling adults recruited at university medical center (Germany)	*n* = 34, 68.8 ± 5.3 (61–80) yrs., 20♀	MRI (T1[3T])	Voxel-wise WB volume	RVR	APOE e4	E4 carriers brainAGE (0.07 yrs) comparable to non-carriers (− 0.67 yrs.; NS)	None
[33]	ADNI study (Longitudinal; US & Canada)	HC: e4+: *n* = 26, 75.0 ± −5.1 yrs.; e4-: *n* = 81, 75.9 ± 4.9 yrs.; sMCI: e4+: *n* = 14, 77.3 ± 5.6 yrs.; e4-: *n* = 22, 76.8 ± 6.5 yrs.; pMCI: e4+: *n* = 78, 74.1 ± 6.5 yrs.; e4-: *n* = 34, 75.5 ± 9.3 yrs.; AD: e4+: *n* = 101, 74.1 ± 6.8 yrs.; e4-: *n* = 49, 75.7 ± 8.9 yrs.; 595-1197 days FU; Sex unknown	MRI (T1[1.5 T])	Voxel-wise GM volume	RVR	APOE e4 carrier status; cognitive function (CDR, ADAS, MMSE)	BrainAGE NS with e4 status at b/line, or FU. Correlates with pMCI cognition at b/line (e4+: CDR & ADAS) & FU (e4+: CDR & ADAS; e4-: ADAS; all *p <* 0.05). AD cognition at b/line (e4+: MMSE; e4-: MMSE, CDR & ADAS) & FU (e4+/−: MMSE, CDR, ADAS; all *p* < 0.05). ^c^Rate differs between e4 groups (~ − 0.01 to 1.68 yrs. per FU yr; *p <* 0.05)	^b^Age, gender
[32]	UK Biobank	Discovery: *n* = 12,378, 46-79 yrs.; Replication: *n* = 4456, 47-80 yrs.; Sex unknown	MRI (T1[3T])	Voxel-based MNI, Jacobian map, GM and WM volume	CNN [Data splitting]	Genetic variance	GAP associated with 2 genetic variants in Discovery (rs2435204-G: ß = 0.11; rs1452628-T: ß = -0.08) & Replication (rs2435204-G: ß = 0.08; rs1452628-T: ß = -0.07; all *p* < 0.01)	^a^Age, age^2^, gender, TICV, 40 PCs, head motion, genotyping, study site

^a^Brain age adjustment; ^b^Model adjustment; ^c^Calculated by regressing time on brain age; *AD* Alzheimer’s Disease; *ADAS* Alzheimer’s; Disease Assessment Score [73]; *ADNI* Alzheimer’s Disease Neuroimaging Initiative; *APOE* Apolipoprotein E genotype; *B/line* Baseline; *BMI* Body mass index; *CDR* Clinical Dementia Rating [75]; *CNN* Convolutional neural networks; *DS* Down Syndrome; *e4+/−* APOE e4 carriers/non-carriers; *FU* Follow-up; *GM* Grey matter; *GPR* Gaussian process regression; *HC* Healthy controls; *IQ* Intellectual quotient; *MMSE* Mini-Mental State Examination [78]; *MNI* Montreal Neurological Institute; *MRI* Magnetic resonance imaging; *NS* Not significant; *NSPN U Change* NEuroScience in Psychiatry Network U-Change project; *PC* Principal-component analysis; *PiB+/−* [11C]-Pittsburgh compound B positive/negative uptake across the brain; *pMCI* Progressive mild cognitive impairment; *PWS* Prader-Willi Syndrome; *RVR* Relevance vector regression; *sMCI* Stable mild cognitive impairment; *SNORD116* Microdelection of SNORD116 gene cluster; *TICV* Total intracranial volume; *WM* White matter; *WB* Whole brain

One genome wide association study using data from the UK Biobank, found and replicated a significant association between brain ageing and two genetic variants - one spanning many genes, including *MAPT*, which encodes for the tau protein (i.e., considered to play a prominent role in Frontotemporal dementia, and other neurodegenerative disorders) [85, 86]; the second is near the *TREK-1* gene, that has been reported (in mice) to play a role in memory impairment, cerebral ischemia, and blood brain barrier dysfunction [87–89].

#### Other factors in ageing populations

Ten studies analysed brain ageing in relation to gender, race, cognitive function, and other measures of biological ageing (i.e., DNA methylation age, telomeres, physical and biological markers of health, and facial ageing) [19, 31], most investigated was cognitive function (Table 6). Six out of seven studies reported a significant association between brain ageing and cognitive function across different domains, most consistent were psychomotor and executive function [31, 32, 53, 54, 90]. The remaining seventh study observed no correlation with working memory, and was the only study to measure cognition via a functional MRI based-task [84].

**Table 6:** Studies investigating gender, race, cognitive function, and other measures of biological ageing

Reference	Study (Design, country)	n, Mean age ± SD (Range), Sex, Other information	Modality (Protocol)	Features	Model (Cross-validation)	Exposure	Main findings outcome	Adjustments
[65]	Cognitively normal, younger/ amyloid negative adults from 6 studies (US)	♀: *n* = 108; ♂: *n* = 76; Age unknown	PET (18F-FDG; 15O-O2, −CO, −H2O)	CMRGIc, CMRO2, CBF, AG	RF [10-fold]	Gender	When trained on ♂ only, GAP ↓ in ♀ (−3.8 yrs., *p <* 0.01). When trained on ♀ only, GAP ↑ in ♂ (+ 2.4 yrs., *p* < 0.04)	^a^Age
[53]	UK Biobank	*n* = 19,000, 10,112♀; Age unknown	MRI (T1, rfMRI, tfMRI, T2 FLAIR, dMRI, swMRI [3 T])	IDP	Non-LR [10-fold]	Gender; cognitive function; imaging features	GAP 0.7 yrs. ↑ in ♀ than ♂. GAP correlates ↓ psychomotor &/or executive function (DSST: *r* = − 0.06; reaction time: *r* = 0.05; TMT-B: *r* = 0.08), fluid intelligence (*r* = − 0.05), & visual memory (pairs matching: *r* = 0.07; all -log_10_P > 8). Correlates with GM & WB volume (r ~ − 0.59 to − 0.49), & microstructure (r ~ 0.25 to 0.41), with differences between sexes (no *p*-value)	^a^Age, age^2^, gender
[19]	Lothian Birth Cohort 1936 (LBC1936) (Scotland)	*n* = 669, 72.7 ± 0.7 yrs., 317♀	MRI (T1[3T])	Voxel-wise WB volume	GPR [10-fold (×1000)]	Gender; MRI; epigenetic clock; TL	♀ PAD (− 1.29 yrs) ↓ than ♂ (4.29 yrs., *p* < 0.001). NS association with epigenetic clock (ρ = − 0.007), or TL (*r* = 0.04). Correlates ↑ CSF, WMH & FA (r ~ 0.27 to 0.49); ↓ GM, WM, CT & MD (r ~ − 0.16 to − 0.47; no p-value)	None
[31]	Dunedin Longitudinal Study (New Zealand)	*n* = 869, 45.2 ± 0.7 (43.5–47.0)yrs.; Cognitive data collected at 3, 7, 9, & 11 yrs. of age; Sex unknown	MRI (T1[3T])	CT, SA & subcortical volume	Stacked RF (SVR)	Adult cognitive function & decline; ageing; brain health at age 3	BA score associated with ↓ total & sub-domains of cognitive function (β ~ − 0.09 to − 0.20) & decline (β ~ − 0.07 to − 0.12; all ***p*** **< 0.05**). ↓ Brain health (β = − 0.12, ***p*** **< 0.05**). ↑ Ageing (pace: β = 0.22; facial: β = 0.15; **both** ***p*** **< 0.05**)	^b^Gender
[84]	Community dwelling adults recruited at university medical center (Germany)	*n* = 34, 68.8 ± 5.3 (61–80) yrs., 20♀	MRI (T1[3T])	Voxel-wise WB volume	RVR	Cognitive function	BranAGE NS correlated with working memory (*r* = 0.01, *p* = 0.98)	None
[90]	Community dwelling adults from DEU [1], CR/RANN [2], & TILDA [3] studies (Turkey, unknown, Ireland)	1) *n* = 175, 69.0 ± 8.6 (47.6–93.5) yrs., 104♀; 2) *n* = 380, 52.4 ± 17.1 (19–80) yrs., 210♀; 3) *n* = 470, 68.6 ± 7.2 (50–88) yrs., 260♀	MRI (T1[1.5/3 T])	Voxel-wise GM density	E-Net [Nested 10-fold]	Cognitive function	1&2) GAP correlates ↓ general cognition (1: ρ = − 0.32; 2: ρ = − 0.14), semantic verbal fluency (1: ρ = − 0.25; 2: ρ = − 0.20) & executive function (TMT-B minus A: 1: ρ ~ 0.12 to 0.27; replicated *p <* 0.05). 1–3) ↓ psychomotor & executive function (TMT-B: ρ ~ 0.09 to 0.27; replicated *p <* 0.05)	^a^Age; ^b^Age, gender
[54]	UK Biobank	*n* = 14,701, 62.6 ± 7.5 yrs., 7914♀	MRI (T1, rfMRI, tfMRI, T2 FLAIR, dMRI, swMRI [3 T])	IDP	LASSO [10-fold]	Cognitive function	↑ GAP associated with ↓ fluid intelligence (B = -0.15), psychomotor &/or executive functions (TMT-B: B = 0.002; tower rearranging: B = -0.12) & non-verbal fluid reasoning (matrix pattern completion: B = -0.22; **all** ***p*** **< 0.001**)	^ab^Age; ^b^age^2^, gender, height, volumetric scaling, & tfMRI head motion
[32]	UK Biobank	Discovery: *n* = 12,378, 46-79 yrs.; Replication: *n* = 4456, 47-80 yrs.; Sex unknown	MRI (T1[3T])	Voxel-based MNI, Jacobian map, GM and WM volume	CNN [Data splitting]	Cognitive function	GAP associated with ↓ psychomotor and/or executive functions (DSST: *r* = − 0.08; reaction time: *r* = 0.03; TMT-A, B, & minus A: r ~ 0.05 to 0.08; **all** ***p*** **< 0.0056**). Fluid intelligence, numeric/prospective/visual memory NS	^a^Age, age^2^, gender, TICV, 40 PCs, head motion, genotyping, study site
[60]	Community dwelling adults from 1 of 6 studies (US)	*n* = 185, 64.9 ± 8.3 yrs., 91♀	MRI (T1[3T])	Voxel-wise WB volume	RVR	Cognitive function	↑ BrainAGE correlates ↓ semantic verbal fluency (*r* = − 0.25, *p* = 0.006)	^b^Age, gender, diabetes duration
[91]	Harvard Ageing Brain Study (US)	AA: *n* = 43, 62-88 yrs., 32♀; Matched NHW: *n* = 43, 65-90 yrs., 30♀; Unmatched NHW: *n* = 43, 64-86 yrs., 29♀	MRI (T1[3T])	CT (Racially different regions in high amyloid group)	SVR [LOO]	Race	GAP −1.05 yrs. for AA & matched NHW; or 0.92 yrs. when unmatched (no p-value). GAP remained when controlling for WMH (AA&Matched: − 0.88 yrs.; Unmatched: − 0.64 yrs.; no p-value)	^a^With/without adjust WMH

Bold = Results corrected for multiple comparisons; ^a^Brain age adjustment; ^b^Model adjustment; *15O-O2/CO/H2O* Oxygen 15 labeled oxygen, carbon dioxide, or water; *18F-FDG* [18F]fluorodeoxyglucose; *AA* African Americans; *AG* Regional aerobic glycolysis; *CBF* Cerebral blood flow; *CMRGlc* Regional total glucose use; *CMRO2* Oxygen consumption; *CNN* Convolutional Neural Networks; *CR/RANN* Cognitive Reserve/Reference Ability Neural Network Study; *CSF* Cerebral spinal fluid; *CT* Cortical thickness; *DEU* Dokuz Eylul University; *dMRI* Diffusion magnetic resonance imaging; *E-Net* Elastic-Net; *FA* Fractional anisotropy; *FLAIR* = T2-weighted fluid-attenuated inversion recovery structural imaging; *GM* Grey matter; *GPR* Gaussian process regression; *IDP* Imaging derived phenotypes (i.e., summary measures of structural and functional brain phenotypes); *LASSO* Least absolute shrinkage and selection operator regression; *LOO* Leave one out; *LR* Linear regression; *MD* Mean diffusivity; *MNI* Montreal Neurological Institute; *MRI* Magnetic resonance imaging; *NHW* Non-Hispanic Whites; *NS* Not significant; *PC* Principal-component analysis; *PET* Positron emission tomography; *RF* Random forest; *RfMRI* Resting state functional magnetic resonance imaging; *RVR* Relevance vector regression; *SA* Surface area; *SwMRI* Suceptibility-weighted imaging; *SVR* Support vector regression; *TfMRI* Task functional magnetic resonance imaging; *TICV* = Total intracranial volume; *TILDA* = The Irish Longitudinal Study of Ageing; *TL* = Telomere length; *TMT* Trail making test ^a^ [92]; *WB* Whole brain; *WM* White matter; *WMH* White matter hyperintensities

Three studies analysed brain ageing using a large sample of participants from the UK Biobank (ranging between 12,378 to 19,000 participants), and reported a significant positive association with a single measure of psychomotor and executive function (i.e., as per the UK Biobank’s Trail Making Task (TMT) B), despite applying different brain age algorithms [32, 53, 54]. However, only one of these three studies reported a significant positive association with all measures from the TMT (TMT-A, −B, and TMT minus B), but included fewer participants [32]. Two of these studies also observed a significant association with complex (i.e., Symbol Digit Substitution Test (DSST)) and simple psychomotor functions (i.e., Reaction time test), while the third had not included these two neuropsychological tests [54]. One additional study, measured brain ageing in three independent cohorts, and reported a significant association with psychomotor and executive function [90]. This same study also reported a significant association for two of the three cohorts that had used the same measure of executive function (i.e., TMT-B minus A) [90]. A fourth study, using longitudinal data (participants were assessed during childhood, and at 45 years of age), found a significant negative association with all measures of adult cognitive function and decline, including psychomotor function (i.e., as per the Wechsler Adult Intelligence Scale-IV, and DSST) [31, 93, 94].

Three studies investigated gender [19, 53, 65]. One study reported decelerated brain ageing for female participants that was significantly lower than the accelerated brain ageing in males [19]. Regardless of whether brain age was trained on males or females only, a second, larger cohort study consistently found decelerated brain ageing in females, that was significantly different to the accelerated brain ageing observed in males [65]. In contrast to these findings, a third study, involving fewer participants (108 and 76 females and males, respectively) estimated non-linear brain age, and found brain ageing in females to be 0.7 years higher than males, though the direction, and significance of this finding remains unclear [53].

Two studies analysed associations with alternative measures of biological ageing [31, 54]. By combining various biological and physical markers (e.g., blood pressure, total cholesterol), Elliot et al. (2019) [31] calculated the pace of ageing and found a significant positive association between this and brain ageing. This same study also reported a significant positive association with subjective measures of facial ageing (i.e., defined by a panel of 8 independent raters) [31]. In contrast, Cole et al. (2020a) [54] found no significant relationship between brain ageing and DNA methylation age (i.e., ‘epigenetic clock’) or telomere length.

### Risk of bias assessment

Details regarding the risk of bias assessment are given in the Additional File 2: Tables S1 to 3. The 35 cohort studies had an overall low risk of bias. The most pertinent sources of potential bias were unclear recruitment and inclusion criteria, not applying or being clear on the methods used to validate brain ageing (the majority of these studies had referenced the validated, Franke et al. (2010) model [55]), and not adjusting for potential confounders. Two, however, had controlled for age or white matter hyperintensities during the development of the brain age model [69, 91]. Three studies included multiple datasets with more than one study design (i.e., cohort and case-control) but had a similar, low level of bias [30, 41, 47]. Of the 18 studies with a case-control design, overall they had higher risk of bias than cohort studies, with the controls not often being comparable to cases (i.e., by confounders, primarily age and sex), and did not identify participants using the same criteria. Further, the method used to measure the exposure/s of interest differed between cases and controls. Only one RCT study design was included and was considered to be of a high quality [63].

## Discussion

This systematic review identified 52 studies which examined the association between genetic, lifestyle, health factors and disease, and brain ageing (age-related changes of the brain defined by the deviation of neuroimaging predicted brain age relative to chronological age). Studies were grouped according to exposure types, with some covering more than one. The majority of evidence on brain ageing came from populations diagnosed with certain forms of mental health or neurological disorders, or cognitive function in normal ageing populations. Evidence regarding the association with lifestyle or environmental, and genetic factors was sparse. Most studies investigated brain ageing in smaller sub-samples of participants drawn from a larger cohort study (34 had one or more samples with less than 100 people) and thus were limited in their statistical sensitivity. Further, some cohorts were a common source of participants for certain exposure types across multiple studies. Inconsistencies were evident for some exposure groups, but were partly attributed to the heterogeneity in study methodologies (i.e., either through design or participant characteristics) or methods of outcome ascertainment.

SZ was the most commonly studied of all exposures, and was consistently shown to be associated with more rapid brain ageing by studies with a relatively low to moderate risk of bias [27, 30, 32, 34, 41, 49, 66]. This is despite methodological differences between studies in terms of the neuroimaging features used to calculate brain ageing, such as cerebral perfusion [27], brain volume and/or density [30, 38, 41, 49, 66] and combinations of cortical thickness, fractional anisotropy, and cognitive performance scores [34]. This corroborates the neuroimaging literature, whereby brain changes overlap those observed in healthy ageing (reductions in brain volume, ventricular enlargement, and cortical thinning) [95–98]. However, concluding mechanisms still vary among studies (i.e., SZ is causative, or the consequence of accelerated ageing), and, in some accounts, limited by the cross-sectional design [27, 32, 34, 38, 41, 49]. Effect sizes vary among studies, and brain ageing in SZ was not always compared to healthy controls. Further, healthy controls deviated from the normal ageing trajectory for some studies, and thus effects may also reflect innate model biases, or the effects of other exposures on brain age prediction.

Evidence of more rapid brain ageing in AD compared to healthy controls was also relatively consistent. Brain atrophy (i.e., the loss of tissue volume) is common with age and is more severe in AD [9]. These findings of accelerated brain ageing corroborate evidence from neuroimaging studies [99, 100], and findings relating to other ageing biomarkers measured in brain tissue [101]. The positive association between brain ageing and disease symptom severity, and the progression from MCI to AD, provides further evidence that AD is directly linked with brain ageing [33, 56, 58, 68]. Findings from two prospective studies also correspond with imaging studies that reported a greater rate of brain atrophy (2% per year for GM volume) in AD patients [33, 56, 102]. An important limitation however, is that all studies of AD used data collected from the ADNI study, and thus, even if the final sample was different between studies, they cannot be considered as entirely independent [33, 56]. Further, the studies only provide a global measure of brain ageing, and thus cannot inform on regional differences in ageing that have been extensively reported in the literature [99, 103, 104].

Evidence across other exposures was relatively inconsistent, in particular with regards to gender and BMI [53, 57]. Heterogeneity in brain age methodologies and participant characteristics are the likely cause of such discrepancies. For example, when investigating gender, two studies reporting preserved ageing in women both used linear models to estimate brain ageing, while the third used a non-linear algorithm, and reported preserved ageing in men. Though this evidence corroborates neuroimaging findings, the literature primarily relates to regional differences (which contrasts the whole brain estimates used by these two eligible studies), and is also relatively inconsistent [65, 105–111]. Further, all three studies had not accounted for potential confounding effects of other environmental exposures, that are specific to certain genders (e.g., education or occupation) and may explain discrepancies between studies, as they have also been associated with altered brain phenotypes [105]. This is a similar limitation when interpreting associations between BMI and brain ageing. BMI is routinely used as a measure of obesity, which is considered to have adverse effects on the brain, and cognitive function in both elderly and SZ populations [112–116]. However, it is attributed to a number of environmental factors (e.g., socioeconomic status, lower physical activity) that may act as confounders in these studies [117]. Study designs and participants varied greatly when investigating BMI as an exposure of brain ageing. Specifically, two of the four studies involved a cohort of older community dwelling participants [53, 57], while the remaining two were case-control studies investigating obesity in young adult populations with SZ [38, 66]. Correlations were only reported by three of four studies investigating BMI, and show little to no relationship with brain ageing. Further, due to the cross-sectional nature of all studies on gender, and BMI, cause and effect relationships could not be determined.

Some studies investigated a number of lifestyle factors, and reported an association between education, physical activity and music with declines in brain ageing [29, 42, 64], while smoking and alcohol consumption were associated with accelerated ageing of the brain [37, 53, 54, 60]. This corroborates the literature, whereby positive lifestyle factors, like physical activity, are associated with preserved structural and functional integrity [118–120], and a reduced risk for AD [121], while smoking and alcohol are found to exacerbate a decline in brain phenotypes [122, 123]. Though this seems promising, the amount of evidence regarding brain ageing is still sparse. Further, studies are cross sectional, and thus temporal and causal relationships cannot be determined. Some studies were also underpowered, while others have limited generalisability (i.e., sampled data from the same cohort study).

Studies used a number of methods to calculate brain ageing. Most common was the framework proposed by Franke et al. (2010) [55] which utilises a relevance vector regression to estimate age from brain volume [29, 33, 36, 38, 41, 42, 44, 49, 50, 55–58, 60, 61, 84]. A large number of studies alternatively used the framework developed by Cole et al. (2015) [51], and thus the second most commonly used algorithm was the gaussian processes regression, primarily when estimating age from brain volume [19, 26, 28, 40, 46, 51, 62, 82, 83]. Considering the contribution by Franke and Cole to the field of brain ageing, the popularity of these frameworks is not surprising. Despite recommendations [124], few studies used multimodal approaches to estimate brain age, which may reflect the popularity of these single modal models; though the need for multiple acquisitions, and greater burden to elderly participants, may have also played a role [34, 53, 54, 67, 69, 125]. Despite a rising interest in deep learning [126, 127], only one study used a convolutional neural network to calculate brain ageing [32].

### Strengths and limitations of review

This systematic review was conducted in accordance with PRISMA guidelines (http://www.prisma-statement.org) [22]. To ensure all relevant publications were included, a systematic search of the brain ageing literature was undertaken, and directed by a registered eligibility criterion, and involved databases and additional literature reviews [20, 21]. Including general and clinical populations increased the coverage exposure types, and thus findings will be of interest to a greater array of research fields. This was also achieved by the inclusion of all neuroimaging modalities and feature types, and reduces any bias towards brain age frameworks that are developed from specific phenotypes (e.g., brain volume, as per Franke et al. (2010) [55] & Cole et al. (2015) [51]).

There are limitations to this systematic review that should be addressed. Considering the contribution of conference papers to machine learning research, the removal of this literary source may have reduced the number of identified papers, and thus influenced the conclusions for this systematic review. The accuracy and generalisability of age prediction were not reported, nor were details regarding the training sample.

### Further directions

This systematic review identified a number of gaps in the brain ageing literature that should be addressed through future research efforts. So far, supervised machine learning is the most popular approach to define brain ageing, particularly when using brain volume as a feature. Comparatively few studies have pursued deep learning approaches to estimating brain ageing. Though they are computationally intensive, there are many benefits that could overcome limitations imposed by other machine learning algorithms, such as the ability to use raw neuroimaging data as input [126, 127]. Clinically, this an appealing option as it is more time efficient (i.e., as no pre-processing is required), and requires little computational engineering [127, 128]. Like deep learning, few studies used multimodal approaches for estimating brain age. Though there are challenges in acquiring, and combining multiple data types; features of various brain phenotypes (obtained from various modalities) could be more informative, and thus may be a more comprehensive approach to investigating brain ageing [125, 129].

Few studies used prospective data, and thus could not investigate cause and effect relationships. Longitudinal studies will help overcome this limitation, and will address questions regarding whether brain age is a biomarker of ageing or disease, thus meeting a key criterion proposed by The American Federation for Ageing Research (i.e., biomarkers must monitor ageing processes, not disease) [130].

The evidence regarding the effects of environmental and lifestyle factors on brain ageing is sparse. Identifying interventions and treatments that are brain preserving, and thus slow the ageing process, is useful knowledge for the ever-growing ageing population, and has many clinical implications, like reducing the strain on age care facilities.

Results regarding brain ageing and gender or BMI were inconsistent. Heterogenous brain ageing methodologies, study designs, and participant characteristics were identified as the likely cause. Thus, to confirm whether findings reflect a true ageing effect, future studies should focus their efforts on replicating these methods, and sampling from populations that are characteristically similar. Information on whether brain ageing is sensitive to gender, or BMI, could help inform certain populations at risk, and be used to prevent poor health outcomes.

Finally, only two eligible studies compared, or combined, brain ageing to alternative ageing biomarkers [19, 31]. It remains unclear whether ageing is tissue specific, or a systematic process, and thus additional knowledge from studies comparing brain ageing with other ageing biomarkers could help resolve this question.

## Conclusion

This systematic review summarised the current evidence for an association between genetic, lifestyle, health, or diseases and brain ageing, the most common being schizophrenia, followed by Alzheimer’s disease. Overall, there is good evidence to suggest schizophrenia is associated with accelerated brain ageing, but limited, or mixed evidence for all other exposures examined. In most cases this was due to a lack of independent replication and consistency across multiple studies that were primarily cross sectional in nature. Thus, future research efforts should focus on replicating current findings, using prospective datasets, to further clarify exposures that may have age preserving, or accelerating properties.

## Supplementary Information

**Additional file 1:.** Completed Prisma 2009 checklist.

**Additional file 2: **Supplementary Results. Risk of bias assessment **Tables S1**–**3**.

## Data Availability

All data generated or analysed during this study are included in this published article and its supplementary information files.

## Abbreviations

AD: Alzheimer’s disease
ADAS: Alzheimer’s disease assessment scale
ADNI: Alzheimer’s disease; neuroimaging initiative
APOE: Apolipoprotein E
BMI: Body mass index
CDR: Clinical dementia rating
HIV: Human immunodeficiency virus
MCI: Mild cognitive impairment
MD: Major depression
MMSE: Mini-mental state examination
MRE: Medical refractory epilepsy
MRI: Magnetic resonance imaging
MS: Multiple sclerosis
NDE: Newly diagnosed focal epilepsy
DSST: Symbol digit substitution test
SZ: Schizophrenia
TMT: Trail making task
TBI: Traumatic brain injury
