# Supplementary material for: Factors associated with brain ageing - a systematic review
Source: BMC Neurol. 2021 Aug 12;21:312. doi: 10.1186/s12883-021-02331-4 (PMC8359541; doi:10.1186/s12883-021-02331-4)
Supplement: Supplementary file 2 — Additional file 2: Supplementary Results. Risk of bias assessment Tables S1–3. [file 12883_2021_2331_MOESM2_ESM.docx]

**SUPPLEMENTARY RESULTS**

**Table S1.** Risk of bias assessment for articles with a cohort study design

| **Reference** | **Group/s from the same population?** | **Recruitment and inclusion criteria clear?** | **Sample characteristics described?** | **Imaging protocol similar?** | **Imaging protocol is clear?** | **Exposure/s measured similar for all groups?** | **Exposure/s measure valid?** | **Outcome/s measure valid?** | **Confounders identified?** | **Strategies to deal with confounders?** | **Appropriate analysis used?** |
| --- | --- | --- | --- | --- | --- | --- | --- | --- | --- | --- | --- |
| (1) | Unclear | No | Yes | Yes | Yes | Unclear | Unclear | No | No | N/A | No |
| (2) | Yes | Yes | Yes | Yes | Yes | Yes | Yes | Yes | No | N/A | Yes |
| (3)  DEU | Yes | Yes | Yes | Yes | Yes | Yes | Yes | Yes | Yes | Yes | Yes |
| (3)  CR/RANN | Yes | Yes | Yes | Yes | Yes | Yes | Yes | Yes | Yes | Yes | Yes |
| (3)  TILDA | Yes | Yes | Yes | Yes | Yes | Yes | Yes | Yes | Yes | Yes | Yes |
| (4) | Yes | Yes | Yes | Yes | Yes | No | Unclear | Yes | Yes | Yes | Yes |
| (5) | Yes | No | Yes | Yes | Yes | Yes | Yes | Yes | No | N/A | Yes |
| (6) | Yes | Yes | Yes | Yes | Yes | Unclear | Unclear | Yes | Yes | Yes | Yes |
| (7) | No | Yes | Yes | No | Yes | Yes | Yes | Yes | Yes | Yes | Yes |
| (8) | Yes | Yes | Yes | Yes | Yes | Yes | Yes | Yes | Yes | Yes | Yes |
| (9) | Yes | Yes | Yes | Yes | Yes | Yes | Yes | Unclear | Yes | Yes | Yes |
| (10) | Yes | Yes | No | Yes | Yes | Yes | Yes | Unclear | Yes | Yes | Yes |
| (11) | Yes | No | Yes | No | Yes | Yes | Yes | Yes | No | N/A | Yes |
| (12) | Yes | Yes | Yes | Yes | Yes | Yes | Yes | No | Yes | Yes | Yes |
| (13) | Yes | Yes | Yes | Yes | Yes | Yes | Yes | No | Yes | Yes | Yes |
| (14) | Yes | No | Yes | Yes | Yes | Yes | Yes | No | Yes | Yes | Yes |
| (15) | Yes | No | Yes | Yes | Yes | No | Yes | No | Yes | Yes | Yes |
| (16) | Yes | No | Yes | Yes | Yes | Yes | Yes | Yes | No | N/A | Yes |
| (17) | Yes | Yes | No | Yes | Yes | Yes | No | Yes | Yes | Yes | Yes |
| (18)(Study 2) | Unclear | Yes | Yes | Yes | Yes | Yes | Yes | Unclear | Yes | Yes | Yes |
| (19) | Yes | Yes | Yes | Yes | Yes | Yes | Yes | No | Yes | Yes | Yes |
| (20) MS (1.5T) | Yes | Yes | Yes | Yes | Yes | Yes | Yes | Yes | Yes | Yes | Yes |
| (21) | No | Yes | Yes | Yes | Yes | Yes | Yes | Yes | No | N/A | Yes |
| (22)  UK Biobank | Yes | Yes | No | Yes | Yes | Yes | Yes | Yes | Yes | Yes | Yes |
| (22)  Icelandic | Yes | Yes | No | Yes | Yes | Unclear | Unclear | Yes | Yes | Yes | Yes |
| (23) | No | Yes | Yes | Yes | Yes | No | Yes | Yes | No | N/A | Yes |
| (24) | Yes | Yes | Yes | Yes | Yes | Yes | Yes | Yes | Yes | Yes | Yes |
| (25) | Yes | No | Yes | Yes | Yes | Yes | Yes | No | Yes | Yes | Yes |
| (26) | Yes | No | Yes | Yes | Yes | Yes | Yes | No | No | N/A | Yes |
| (27) | Yes | Yes | Yes | Yes | Yes | N/A | N/A | Yes | Yes | Yes | No |
| (28) | No | Yes | Yes | Yes | Yes | Yes | Yes | Yes | Yes | Yes | Yes |
| (29) | Yes | No | Yes | Yes | Yes | Yes | Yes | Yes | Yes | Yes | Yes |
| (30) | Yes | Yes | Yes | Yes | Yes | Yes | Yes | No | No | N/A | Yes |
| (31)  Sample 1 & 2 | No | No | No | Yes | Yes | Yes | Yes | Yes | Yes | Yes | Yes |
| (32) | Yes | Yes | No | No | Yes | Yes | Yes | No | No | N/A | Yes |
| (33) | Yes | No | No | Yes | Yes | Yes | Unclear | Yes | Yes | Yes | No |
| (34) | Yes | Yes | Yes | Yes | Yes | Yes | Yes | Yes | Yes | Yes | Yes |
| (35) | Yes | Yes | Yes | Yes | Yes | Yes | Yes | Unclear | Yes | Yes | Yes |

**Table S2.** Risk of bias assessment for articles with a case-control study design

| **Reference** | **Cases & controls comparable?** | **Cases & controls matched appropriately?** | **Identified using the same criteria?** | **Sample characteristics described?** | **Imaging protocol is similar?** | **Imaging protocol is clear?** | **Exposure/s measure is valid?** | **Exposure/s measured similarly for cases & controls?** | **Exposure period long enough?** | **Outcome/s measure valid?** | **Confounding factors identified?** | **Strategies to deal with confounders?** | **Appropriate analysis used?** |
| --- | --- | --- | --- | --- | --- | --- | --- | --- | --- | --- | --- | --- | --- |
| (36)  Sample 1 | Yes | Yes | Yes | Yes | Yes | Yes | Yes | Yes | Yes | Yes | Yes | Yes | Yes |
| (36)  Sample 2 | No | No | No | Yes | Yes | Yes | Yes | Unclear | Yes | Yes | No | N/A | No |
| (37) | Yes | Yes | Yes | Yes | Yes | Yes | Yes | Yes | Yes | No | No | N/A | Yes |
| (38) | Yes | Yes | Yes | Yes | Yes | Yes | Yes | Yes | Yes | Yes | Yes | Yes | Yes |
| (39) | No | No | No | Yes | Yes | Yes | Yes | Yes | Yes | Yes | Yes | Yes | Yes |
| (40) | Unclear | Unclear | Unclear | Yes | Yes | Yes | Yes | Unclear | Yes | Yes | No | N/A | Yes |
| (41) | No | Yes | Yes | Yes | Yes | Yes | Yes | Yes | Yes | Yes | Yes | Yes | Yes |
| (18) (Study 1) | Yes | Yes | Yes | Yes | Yes | Yes | Yes | Yes | Yes | Unclear | Yes | Yes | Yes |
| (42) | No | No | Yes | Yes | No | Yes | Yes | Unclear | Yes | Yes | No | N/A | Yes |
| (20) MS & HC (3T) | Yes | Yes | No | Yes | Yes | Yes | Yes | No | Yes | Yes | Yes | Yes | Yes |
| (43) | No | No | No | Yes | Yes | Yes | Yes | Yes | Yes | No | Yes | Yes | Yes |
| (44) | Yes | Yes | Yes | Yes | Yes | Yes | Yes | Yes | Yes | Yes | Yes | Yes | Yes |
| (45) | No | Yes | Yes | Yes | Yes | Yes | Unclear | No | Yes | No | Yes | Yes | Yes |
| (46) | No | Yes | Unclear | Yes | Yes | Yes | Yes | No | Yes | No | No | N/A | Yes |
| (47) | Yes | No | No | Yes | Yes | Yes | Yes | No | Yes | No | Yes | Yes | Yes |
| (48) | No | No | No | Yes | Yes | Yes | Yes | No | Yes | Yes | Yes | Yes | Yes |
| (49) | Yes | Yes | Yes | Yes | Yes | Yes | No | Yes | Yes | No | No | N/A | Yes |
| (31) Sample 4 | Unclear | Yes | No | No | Yes | Yes | Yes | No | Yes | Yes | No | N/A | No |
| (50) | No | No | No | Yes | Yes | Yes | Yes | No | Yes | Yes | Yes | Yes | Yes |
| (51) | No | No | Yes | Yes | Yes | Yes | Yes | Yes | Yes | Yes | Yes | Yes | Yes |

**Table S3.** Risk of bias assessment for the one article using a randomised control trial study design

| **Reference** | **Sample characteristics described?** | **Imaging protocol is clear?** | **Randomisation assigned participants?** | **Treatment groups similar at baseline?** | **Randomisation was double-blinded?** | **Groups treated identically other than the intervention of interest?** | **Follow up complete?** | **Participants analysed in randomised group** | **Outcome/s measured similarly?** | **Outcome/s measure valid?** | **Appropriate analysis was used?** |
| --- | --- | --- | --- | --- | --- | --- | --- | --- | --- | --- | --- |
| (52) | Yes | Yes | Yes | Yes | Yes | Yes | Yes | Yes | Yes | Yes | Yes |

**REFERENCES**

1. Amen DG, Egan S, Meysami S, Raji CA, George N. Patterns of Regional Cerebral Blood Flow as a Function of Age Throughout the Lifespan. J Alzheimers Dis. 2018;65:1087-92.

2. Beheshti I, Maikusa N, Matsuda H. The association between "Brain-Age Score" (BAS) and traditional neuropsychological screening tools in Alzheimer's disease. Brain Behav. 2018;8:e01020.

3. Boyle R, Jollans L, Rueda-Delgado LM, Rizzo R, Yener GG, McMorrow JP, et al. Brain-predicted age difference score is related to specific cognitive functions: a multi-site replication analysis. Brain Imaging Behav. 2020.

4. Cole JH, Underwood J, Caan MWA, De Francesco D, Van Zoest RA, Leech R, et al. Increased brain-predicted aging in treated HIV disease. Neurology. 2017b;88:1349-57.

5. Cole JH, Ritchie SJ, Bastin ME, Valdes Hernandez MC, Munoz Maniega S, Royle N, et al. Brain age predicts mortality. Mol Psychiatry. 2018;23:1385-92.

6. Cole JH. Multimodality neuroimaging brain-age in UK biobank: relationship to biomedical, lifestyle, and cognitive factors. Neurobiol Aging. 2020a;92:34-42.

7. Cole JH, Raffel J, Friede T, Eshaghi A, Brownlee WJ, Chard D, et al. Longitudinal assessment of multiple sclerosis with the brain-age paradigm. Ann Neurol. 2020b;88:93-105.

8. Cruz-Almeida Y, Fillingim RB, Riley JL, Woods AJ, Porges E, Cohen R, et al. Chronic pain is associated with a brain aging biomarker in community-dwelling older adults. Pain. 2019;160:1119-30.

9. Egorova N, Liem F, Hachinski V, Brodtmann A. Predicted Brain Age After Stroke. Front Aging Neurosci. 2019;11:348.

10. Elliott ML, Belsky DW, Knodt AR, Ireland D, Melzer TR, Poulton R, et al. Brain-age in midlife is associated with accelerated biological aging and cognitive decline in a longitudinal birth cohort. Mol Psychiatry. 2019.

11. Franke K, Ziegler G, Kloppel S, Gaser C, Alzheimer's Disease Neuroimaging I. Estimating the age of healthy subjects from T1-weighted MRI scans using kernel methods: exploring the influence of various parameters. Neuroimage. 2010;50:883-92.

12. Franke K, Gaser C. Longitudinal changes in individual BrainAGE in healthy aging, mild cognitive impairment, and Alzheimer’s disease. GeroPsych. 2012;25:235.

13. Franke K, Gaser C, Manor B, Novak V. Advanced BrainAGE in older adults with type 2 diabetes mellitus. Front Aging Neurosci. 2013;5:90.

14. Franke K, Ristow M, Gaser C. Gender-specific impact of personal health parameters on individual brain aging in cognitively unimpaired elderly subjects. Front Aging Neurosci. 2014;6:94.

15. Franke K, Hagemann G, Schleussner E, Gaser C. Changes of individual BrainAGE during the course of the menstrual cycle. Neuroimage. 2015;115:1-6.

16. Gaser C, Franke K, Kloppel S, Koutsouleris N, Sauer H. BrainAGE in Mild Cognitive Impaired Patients: Predicting the Conversion to Alzheimer's Disease. PLoS ONE. 2013;8:e67346.

17. Goyal MS, Blazey TM, Su Y, Couture LE, Durbin TJ, Bateman RJ, et al. Persistent metabolic youth in the aging female brain. Proc Natl Acad Sci U S A. 2019;116:3251-5.

18. Hajek T, Franke K, Kolenic M, Capkova J, Matejka M, Propper L, et al. Brain Age in Early Stages of Bipolar Disorders or Schizophrenia. Schizophr Bull. 2019;45:190-8.

19. Hatton SN, Franz CE, Elman JA, Panizzon MS, Hagler DJ, Jr., Fennema-Notestine C, et al. Negative fateful life events in midlife and advanced predicted brain aging. Neurobiol Aging. 2018;67:1-9.

20. Hogestol EA, Kaufmann T, Nygaard GO, Beyer MK, Sowa P, Nordvik JE, et al. Cross-sectional and longitudinal MRI brain scans reveal accelerated brain aging in multiple sclerosis. Front Neurol. 2019;10:450.

21. Hwang G, Hermann B, Nair VA, Conant LL, Dabbs K, Mathis J, et al. Brain aging in temporal lobe epilepsy: Chronological, structural, and functional. NeuroImage Clin. 2020;25:102183.

22. Jonsson BA, Bjornsdottir G, Thorgeirsson TE, Ellingsen LM, Walters GB, Gudbjartsson DF, et al. Brain age prediction using deep learning uncovers associated sequence variants. Nat Commun. 2019;10:5409.

23. Koutsouleris N, Davatzikos C, Borgwardt S, Gaser C, Bottlender R, Frodl T, et al. Accelerated brain aging in schizophrenia and beyond: a neuroanatomical marker of psychiatric disorders. Schizophr Bull. 2014;40:1140-53.

24. Liem F, Varoquaux G, Kynast J, Beyer F, Kharabian Masouleh S, Huntenburg JM, et al. Predicting brain-age from multimodal imaging data captures cognitive impairment. Neuroimage. 2017;148:179-88.

25. Lowe LC, Gaser C, Franke K. The effect of the APOE genotype on individual BrainAGE in normal aging, Mild cognitive impairment, and Alzheimer's Disease. PLoS ONE. 2016;11:e0157514.

26. Luders E, Gingnell M, Poromaa IS, Engman J, Kurth F, Gaser C. Potential Brain Age Reversal after Pregnancy: Younger Brains at 4-6Weeks Postpartum. Neuroscience. 2018;386:309-14.

27. McDonough IM. Beta-amyloid and Cortical Thickness Reveal Racial Disparities in Preclinical Alzheimer's Disease. Neuroimage Clin. 2017;16:659-67.

28. Richard G, Kolskar K, Ulrichsen KM, Kaufmann T, Alnaes D, Sanders AM, et al. Brain age prediction in stroke patients: Highly reliable but limited sensitivity to cognitive performance and response to cognitive training. NeuroImage Clin. 2020;25:102159.

29. Savjani RR, Taylor BA, Acion L, Wilde EA, Jorge RE. Accelerated Changes in Cortical Thickness Measurements with Age in Military Service Members with Traumatic Brain Injury. J Neurotrauma. 2017;34:3107-16.

30. Scheller E, Schumacher LV, Peter J, Lahr J, Wehrle J, Kaller CP, et al. Brain aging and APOE epsilon4 interact to reveal potential neuronal compensation in healthy older adults. Front Aging Neurosci. 2018;10:74.

31. Schnack HG, Van Haren NEM, Nieuwenhuis M, Pol HEH, Cahn W, Kahn RS. Accelerated brain aging in schizophrenia: A longitudinal pattern recognition study. Am J Psychiatry. 2016;173:607-16.

32. Shahab S, Mulsant BH, Levesque ML, Calarco N, Nazeri A, Wheeler AL, et al. Brain structure, cognition, and brain age in schizophrenia, bipolar disorder, and healthy controls. Neuropsychopharmacology. 2019;44:898-906.

33. Smith SM, Vidaurre D, Alfaro-Almagro F, Nichols TE, Miller KL. Estimation of brain age delta from brain imaging. NeuroImage. 2019;200:528-39.

34. Steffener J, Habeck C, O'Shea D, Razlighi Q, Bherer L, Stern Y. Differences between chronological and brain age are related to education and self-reported physical activity. Neurobiol Aging. 2016;40:138-44.

35. Underwood J, Cole JH, Leech R, Sharp DJ, Winston A, group C. Multivariate Pattern Analysis of Volumetric Neuroimaging Data and Its Relationship With Cognitive Function in Treated HIV Disease. J Acquir Immune Defic Syndr. 2018;78:429-36.

36. Azor AM, Cole JH, Holland AJ, Dumba M, Patel MC, Sadlon A, et al. Increased brain age in adults with Prader-Willi syndrome. Neuroimage Clin. 2019;21:101664.

37. Besteher B, Gaser C, Nenadic I. Machine-learning based brain age estimation in major depression showing no evidence of accelerated aging. Psychiatry Res Neuroimaging. 2019;290:1-4.

38. Chen CL, Shih YC, Liou HH, Hsu YC, Lin FH, Tseng WYI. Premature white matter aging in patients with right mesial temporal lobe epilepsy: A machine learning approach based on diffusion MRI data. NeuroImage Clin. 2019;24:102033.

39. Cole JH, Leech R, Sharp DJ. Prediction of brain age suggests accelerated atrophy after traumatic brain injury. Ann Neurol. 2015;77:571-81.

40. Cole JH, Annus T, Wilson LR, Remtulla R, Hong YT, Fryer TD, et al. Brain-predicted age in Down syndrome is associated with beta amyloid deposition and cognitive decline. Neurobiol Aging. 2017c;56:41-9.

41. Guggenmos M, Schmack K, Sekutowicz M, Garbusow M, Sebold M, Sommer C, et al. Quantitative neurobiological evidence for accelerated brain aging in alcohol dependence. Transl Psychiatry. 2017;7:1279.

42. Han LKM, Dinga R, Hahn T, Ching CRK, Eyler LT, Aftanas L, et al. Brain aging in major depressive disorder: results from the ENIGMA major depressive disorder working group. Mol Psychiatry. 2020.

43. Kolenic M, Franke K, Hlinka J, Matejka M, Capkova J, Pausova Z, et al. Obesity, dyslipidemia and brain age in first-episode psychosis. J Psychiatr Res. 2018;99:151-8.

44. Kuhn T, Kaufmann T, Doan NT, Westlye LT, Jones J, Nunez RA, et al. An augmented aging process in brain white matter in HIV. Hum Brain Mapp. 2018;39:2532-40.

45. Luders E, Cherbuin N, Gaser C. Estimating brain age using high-resolution pattern recognition: Younger brains in long-term meditation practitioners. Neuroimage. 2016;134:508-13.

46. Moeller JR, Eidelberg D. Divergent expression of regional metabolic topographies in Parkinson's disease and normal ageing. Brain. 1997;1:2197-206.

47. Nenadic I, Dietzek M, Langbein K, Sauer H, Gaser C. BrainAGE score indicates accelerated brain aging in schizophrenia, but not bipolar disorder. Psychiatry Res. 2017;266:86-9.

48. Pardoe HR, Cole JH, Blackmon K, Thesen T, Kuzniecky R. Structural brain changes in medically refractory focal epilepsy resemble premature brain aging. Epilepsy Res. 2017;133:28-32.

49. Rogenmoser L, Kernbach J, Schlaug G, Gaser C. Keeping brains young with making music. Brain Struct Funct. 2018;223:297-305.

50. Sone D, Beheshti I, Maikusa N, Ota M, Kimura Y, Sato N, et al. Neuroimaging-based brain-age prediction in diverse forms of epilepsy: a signature of psychosis and beyond. Mol Psychiatry. 2019.

51. Van Gestel H, Franke K, Petite J, Slaney C, Garnham J, Helmick C, et al. Brain age in bipolar disorders: Effects of lithium treatment. Aust N Z J Psychiatry. 2019. 53:1179-88.

52. Le TT, Kuplicki R, Yeh HW, Aupperle RL, Khalsa SS, Simmons WK, et al. Effect of Ibuprofen on BrainAGE: A Randomized, Placebo-Controlled, Dose-Response Exploratory Study. Biol Psychiatry Cogn Neurosci Neuroimaging. 2018;3:836-43.
